# Taxonomy and Distribution of Freshwater Pearl Mussels (Unionoida: Margaritiferidae) of the Russian Far East

**DOI:** 10.1371/journal.pone.0122408

**Published:** 2015-05-26

**Authors:** Ivan N. Bolotov, Yulia V. Bespalaya, Ilya V. Vikhrev, Olga V. Aksenova, Paul E. Aspholm, Mikhail Y. Gofarov, Olga K. Klishko, Yulia S. Kolosova, Alexander V. Kondakov, Artyom A. Lyubas, Inga S. Paltser, Ekaterina S. Konopleva, Sakboworn Tumpeesuwan, Nikita I. Bolotov, Irina S. Voroshilova

**Affiliations:** 1 Institute of Ecological Problems of the North of the Ural Branch of the Russian Academy of Sciences, Arkhangelsk, Russia; 2 Norwegian Institute for Agricultural and Environmental Research (Bioforsk), Svanhovd, Svanvik, Norway; 3 Institute of Natural Resources, Ecology and Cryology of the Siberian Branch of the Russian Academy of Sciences, Chita, Russia; 4 Department of Biology, Faculty of Science, Maha Sarakham University, Maha Sarakham, Thailand; 5 I.D. Papanin Institute of the Biology of Inland Waters of the Russian Academy of Sciences, Yaroslavl oblast, Nekouzsky district, Borok, Russia; Australian Museum, AUSTRALIA

## Abstract

The freshwater pearl mussel family Margaritiferidae includes 13 extant species, which are all listed by IUCN as endangered or vulnerable taxa. In this study, an extensive spatial sampling of *Margaritifera* spp. across the Russian Far East (Amur Basin, Kamchatka Peninsula, Kurile Archipelago and Sakhalin Island) was conducted for a revision of their taxonomy and distribution ranges. Based on their DNA sequences, shell and soft tissue morphology, three valid species were identified: *Margaritifera dahurica* (Middendorff, 1850), *M*. *laevis* (Haas, 1910) and *M*. *middendorffi* (Rosén, 1926). *M*. *dahurica* ranges across the Amur basin and some of the nearest river systems. *M*. *laevis* is distributed in Japan, Sakhalin Island and the Kurile Archipelago. *M*. *middendorffi* was previously considered an endemic species of the Kamchatka. However, it is widespread in the rivers of Kamchatka, Sakhalin Island, the Kurile Islands (across the Bussol Strait, which is the most significant biogeographical boundary within the archipelago), and, likely, in Japan. The Japanese species *M*. *togakushiensis* Kondo & Kobayashi, 2005 seems to be conspecific with *M*. *middendorffi* because of similar morphological patterns, small shell size (<100 mm long) and overlapped ranges, but it is in need of a separate revision. Phylogenetic analysis reveals that two NW Pacific margaritiferid species, *M*. *laevis* and *M*. *middendorffi*, formed a monophyletic 18S rDNA clade together with the North American species *M*. *marrianae* and *M*. *falcata*. The patterns that were found in these *Margaritifera* spp. are similar to those of freshwater fishes, indicating multiple colonizations of Eastern Asia by different mitochondrial lineages, including an ancient Beringian exchange between freshwater faunas across the Pacific.

## Introduction

The family Margaritiferidae includes 13 extant species, which are mainly distributed in temperate latitudes of the Northern hemisphere [1], [2], [3]. Smith [4] provided a detailed diagnosis of the family. Recent species are known from North America, Europe, Northern Africa, the Middle East, and throughout much of Southern and Eastern Asia [2], [4]. The most ancient Margaritiferidae fossils are known from the Upper Triassic and Lower Jurassic fluvio-lacustrine deposits in the Sichuan, Southeastern China [5], [6]. The recent margaritiferids retain the simple, unfused mantle margins from the ancestral palaeoheterodont and several other ‘plesiomorphic’ features; therefore, these species have been regarded as the basal unionoid family [3], [7].

The North American, European and Northern African Margaritiferidae are relatively well studied [8], [4], [9], [10], [11] in contrast to the Asian representatives of the family. Most of the references for Far Eastern freshwater pearl mussel populations are from Japan [12], [13], [14], [15], [16]. Recently, a description of a new Japanese Margaritiferidae species *M*. *togakushiensis* Kondo & Kobayashi, 2005 was published. This species was separated from *M*. *laevis* based on the results of long-term studies, specifically including differences in the host fish preference and genetic and morphological patterns [17], [18], [19], [20], [21].

Reliable data on the Far Eastern Russian Margaritiferidae are relatively poor. Several old studies contain most of the available information [22], [23], [24], [25]. Unfortunately, later taxonomic revisions of the Russian Margaritiferidae are based on the so-called comparatory method that uses a single character to differentiate species, the frontal curvature of the shell [26], [27], [28], [29], [30]. This method does not account for the simple axiom about bivalve shell flexibility that shell shape has intraspecific variation according to environmental gradients [31], [32], [33], [34]. Therefore, any works that are based on the comparatory method usually cannot be used as reliable references for mussel taxonomy, although they provide a wide variety of material for species synonymy. For example, five comparatory Margaritiferidae “taxa” from the Amur Basin, were described [26], [28], [29]. Some authors published interesting data about the reproductive biology of *M*. *dahurica* and its relationships with bitterlings in Transbaikalia [35], [36]. Important studies of the Russian Margaritiferidae [8] clarified their taxonomy and provided some information on population size and biology, but ignored the comparatory method. Akiyama et al. [37] presented data with respect to two recorded *Margaritifera* species on Sakhalin Island.

Smith [4] summarized the available data for all of the Margaritiferidae species, including the Asian representatives, and revised the family based on morphological and anatomical data. In addition, Smith conducted an analysis of each species’ distribution using relatively detailed range maps. An analysis of the molecular phylogeny of Margaritiferidae shows that an ancient history of this Laurasian family is very difficult to interpret [10], [38]. Referenced authors have associated the problems with a pattern of extinction and contraction of an ancient Margaritiferidae fauna with the peripheral isolation of a formerly widespread taxon, fish host dispersal or even host switching. In cited papers, the sequences of only two Asian species (*M*. *dahurica* and *M*. *laevis*) were used. The Margaritiferidae taxonomy that was proposed by Smith [4], was not confirmed by recent molecular data [10], [38], [39].

Thus, the available information on the Asian freshwater pearl mussels is limited. Their taxonomy is not completely clear and reliable data on the species’ ranges are sparse. However, freshwater pearl mussels are extremely demanding regarding habitat quality and can exist only in a narrow range of environmental conditions [11]. The majority of these species are listed by IUCN as endangered or vulnerable taxa [8], [9], [10], [11], [40]. Freshwater mussels have experienced one of the highest rates of extinction of any group of organisms in the past 100 years [41]. Therefore, reliable information on the taxonomy and distribution ranges is still needed for the conservation of the *Margaritifera* species.

Based on the most spatially comprehensive sampling of the Margaritiferidae that has ever been conducted in the Far Eastern rivers, we here address three questions concerning the taxonomy and distribution of these species:

1. How many species are living in the Far Eastern Russian rivers? What are their recent detailed ranges?
2. What are the phylogenetic relationships of the Margaritiferidae from the Russian Far East?
3. Is it possible to obtain a reliable identification of these species by using a morphological pattern?

## Methods

### Sampling

**Fig 1:**
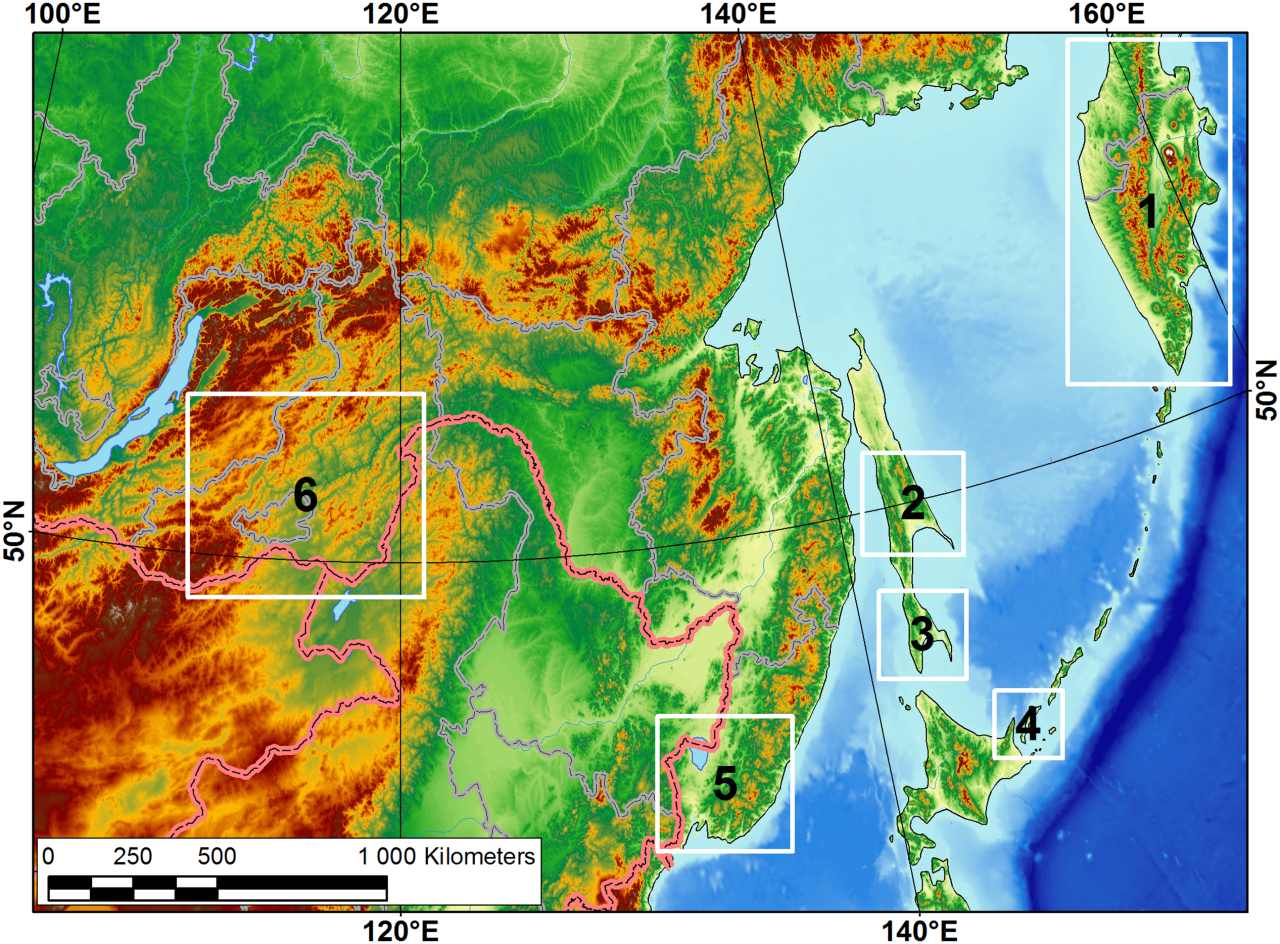
Map of location of the field study areas. 1—Kamchatka Peninsula (2012, I. Bolotov, Y. Bespalaya, I. Vikhrev, M. Gofarov), 2—Central Sakhalin (2012, same team), 3—southern Sakhalin (2011–2012, same team & Y. Kolosova, O. Aksenova), 4—Kunashir Island (2011, same team & Y. Kolosova, O. Aksenova), 5—Primorye (2012, same team), 6—Transbaikalia (2004–2011, O.K. Klishko). Data on the studied river sites are presented in S1–S4 Tables.

We conducted field studies within several regions of Eastern Russia in the period 2004–2012 (see Fig 1 for the location of the study areas). Sampling on the Russian Federation territory was permitted within the framework of the special grants of the Russian Foundation for Basic Research (RFBR, no. 12-04-00594), the Ministry of Science and Education of Russia (no. № P362), and the scientific program of the Ural Branch of Russian Academy of Sciences (no. 12-P-5-1014). Sampling on the protected sites of the Kunashir Island was permitted by the Directorate of the State Nature Protected Area “Kurilsky” (scientific agreement no. 5/11 of 17.08/2010 between the Institute of Ecological Problems of the North of the Ural Branch of Russian Academy of Sciences and the Kurilsky Nature Reserve).

For molecular analyses, the tissue samples were collected from 52 live bivalves in 15 localities (see S1 Table). Samples were immediately preserved in 96% ethanol. Dead shells were collected from each field site for morphological investigation; additional shells from some museum collections were studied (see S2–S4 Tables). A total of 554 shells from 44 localities were studied (426 from our collection and 128 museum specimens).

### Morphological studies

The total length of the live specimens and dead shells were measured to the nearest 0.1 mm with dial calipers [8]. The comparative morphologies of the shells were analyzed using shape and structure of the pseudo-cardinal and lateral teeth in the valves, shell shape, umbo position and the patterns of distribution of mantle attachment scars on the inner shell surface. We calculated the plane projection squares of the inner surface of the right valve for the estimation of the density of mantle attachment scars. Ten specimens of each species were taken for analysis. The squares were calculated using SHAPE v.1.3 software [42]. The scars were counted by the GNU Image Manipulation Program (GIMP) v.2.8. A normality test of the obtained density values and a one-way ANOVA Welsh’s test (S5 Table) were calculated using STATISTICA v.10 software.

The soft tissue morphologies were analyzed and compared based on the shape and structure of the inhalant siphon, gills and labial palps. Shell and soft tissue photos were taken using a DSLR camera (Canon EOS 7D, Japan) with a 24–70 mm lens (Sigma AF 24–70 mm f/2.8 IF EX DG ASPHERICAL HSM, Japan), and photos of the shell structure details and inhalant siphons were taken using a stereomicroscope (Leica M2C, Germany) and a DSLR camera with a 100 mm macro lens (Canon Macro Lens EF 100 mm 1:2,8 L IS USM, Japan). Descriptions of the shell morphologies were based on an analysis of the collected specimens and museum samples using the original species descriptions [18], [38], [43], [44], [45], [46] and a few other works [23], [24], [47], [48].

We studied the type specimens for the following taxa: *Unio* (*Alasmodonta*) *dahuricus* Middendorff, 1850; *Margaritana middendorffi* Rosén, 1926 (including specimens of *Unio* (*Alasmodonta*) *complanatus* Middendorff, 1851); and *M*. *sachalinensis* Zhadin, 1938 (ZISP). The type series of several comparatory “taxa” were also investigated, including *Dahurinaia sujfunensis* Moskvicheva, 1973; *D*. *kurilensis* Zatravkin & Starobogatov, 1984; *D*. *tiunovae* Bogatov & Zatravkin, 1988; *D*. *komarovi* Bogatov et al., 2003; *D*. *ussuriensis* Bogatov et al., 2003; *D*. *prozorovae* Bogatov et al., 2003; *D*. *transbaicalica* Klishko, 2008; *Kurilinaia kamchatica* Bogatov et al., 2003; and *K*. *zatravkini* Bogatov et al., 2003 (ZISP). In general, the ZISP systematic catalog has a very complicated numeration because V. V. Bogatov has transferred many specimens from different samples, including true type series, to his newly described comparatory “taxa”.

### Species range mapping

The mapping was based on the data of our field studies (see S2–S4 Tables). We determined the precise coordinates of investigated river sites using a geographical positioning system (GPS). Data on other localities were obtained from museum collections and reliable published studies (see S2–S4 Tables). In the cases where a particular map scale did not allow separated precision pointing of closely situated localities, we marked those localities one by one with points and joined them under the one number in the respective table. The arrangement of the localities was digitized and mapped using ESRI ArcGIS 10. The presumed error of determination of the locality coordinates is around ±1–2 km, because published records and collection labels are usually ascribed to an approximate location. The layers of digital maps were added from standard ESRI Data & Maps 10 dataset.

### PCR amplification and DNA sequencing

The present study includes new molecular data for 53 Margaritiferidae specimens (S1 Table). Genomic DNA was extracted from the foot or mantle tissues using the Diatom DNA Prep 200 reagents kit (“Laboratoriya Isogen” LLC, Russia) following the manufacturer’s protocol. The standard forward primer LCO 1490 [49] was used to amplify the mitochondrial COI gene from every species. As reverse COI primers, HCO 2198 in a modified version (without the first 6 bases at the 5´ end) was applied to *M*. *dahurica*, and C1-N-2329 was used for two other Margaritiferidae species [49], [50]. The newly designed primers 18S-IF (5´-TTCCTTAGATCGTACAATCCTAC-3´) and 18S-IR (5´-TCCTATTCCATTATTCCATGC-3´) were used to amplify the nuclear 18S rRNA gene from every species. The PCR mix contained approximately 200 ng of total cellular DNA, 10 pmol of each primer, 200 μmol of each dNTP, 2.5 μl of PCR buffer (with 10×2 mmol MgCl_2_), 0.8 units of Taq DNA polymerase (SibEnzyme Ltd., Novosibirsk, Russia), and H_2_O, which was added up to a final volume of 25 μl. Thermocycling included one cycle at 95°C (4 min), followed by 30–35 cycles of 95°C (50 sec), 52°C (50 sec), and 72°C (50 sec) and a final extension at 72°C (5 min). Forward and reverse sequencing was performed on an automatic sequencer (ABI PRISM 3730, Applied Biosystems) using the ABI PRISM BigDye Terminator v. 3.1 reagent kit. The resulting sequences were checked using a sequence alignment editor (BioEdit version 7.2.5, [51], [52]). In addition, 25 sequences were obtained from NCBI’s GenBank, including two sequences of *Unio pictorum* as outgroup (S6 Table).

### Sequence alignment and phylogenetic analyses

The alignment of the COI and 18S sequences was performed using the ClustalW algorithm implemented in MEGA6 [53]. For the phylogenetic analyses, each sequence of the aligned datasets was trimmed, leaving a 654-bp COI and a 681-bp 18S fragment. The sequence datasets were collapsed into haplotypes using an online FASTA sequence toolbox (FaBox 1.41, [54]). The analyses were performed using 24 COI and 8 18S unique haplotypes, including an outgroup. The GTR+I model of sequence evolution was used for the COI gene sequences, and the K2+I model was used for the 18S gene sequences based on the corrected Akaike Information Criterion for small sample sizes (AICc) in MEGA6 [53]. Phylogenetic relationships were reconstructed for each gene separately, based on Bayesian inference as implemented in the software package MrBayes version 3.2.2 [55]. Four Markov chains, one cold and three heated (temperature = 0.1), were run simultaneously for 1,000,000 generations. The trees were sampled every 100^th^ generation. After completing the Markov Chain Monte Carlo (MCMC) analysis, the first 2,500 trees (25%) were discarded as burn-in, and the majority-rule consensus tree was calculated from the remaining 7,500 trees. The convergence of the MCMC chains to a stationary distribution was checked visually based on the plotted posterior estimates using an MCMC trace analysis tool (Tracer version 1.6, [56]). The effective sample size (ESS) for each parameter that was sampled from the MCMC analysis was observed to be greater than 1000. The combined set of trees showed a smooth frequency plot. The resulting phylogenies were constructed using a tree figure drawing tool (FigTree software version 1.4.0 [57]).

### Nomenclatural acts

The electronic edition of this article conforms to the requirements of the amended International Code of Zoological Nomenclature, and hence the new names contained herein are available under that Code from the electronic edition of this article. This published work and the nomenclatural acts it contains have been registered in ZooBank, the online registration system for the ICZN. The ZooBank LSIDs (Life Science Identifiers) can be resolved and the associated information viewed through any standard web browser by appending the LSID to the prefix “http://zoobank.org/”. The LSID for this publication is: urn:lsid:zoobank.org:pub: 46C93315-18E0-45DD-B121-1F225D6E200F. The electronic edition of this work was published in a journal with an ISSN, and has been archived and is available from the following digital repositories: PubMed Central and LOCKSS.

### Deposition of examined material

RMBH—Russian Museum of the Biodiversity Hotspots of Institute of Ecological Problems of the North of the Ural Branch of Russian Academy of Science, Arkhangelsk, 163000, Russia.

INREC—Institute of Natural Resources, Ecology and Cryology of the Siberian Branch of Russian Academy of Sciences, Chita, 672000, Russia.

ZISP—Zoological Institute of the Russian Academy of Sciences, St. Petersburg, 199034, Russia.

SMF—Forschungsinstitut und Natur-Museum Senckenberg, Senckenberg-Anlage 25, 6000 Frankfurt-am-Main 1, Germany.

## Results

### Phylogenetic analyses

**Fig 2:**
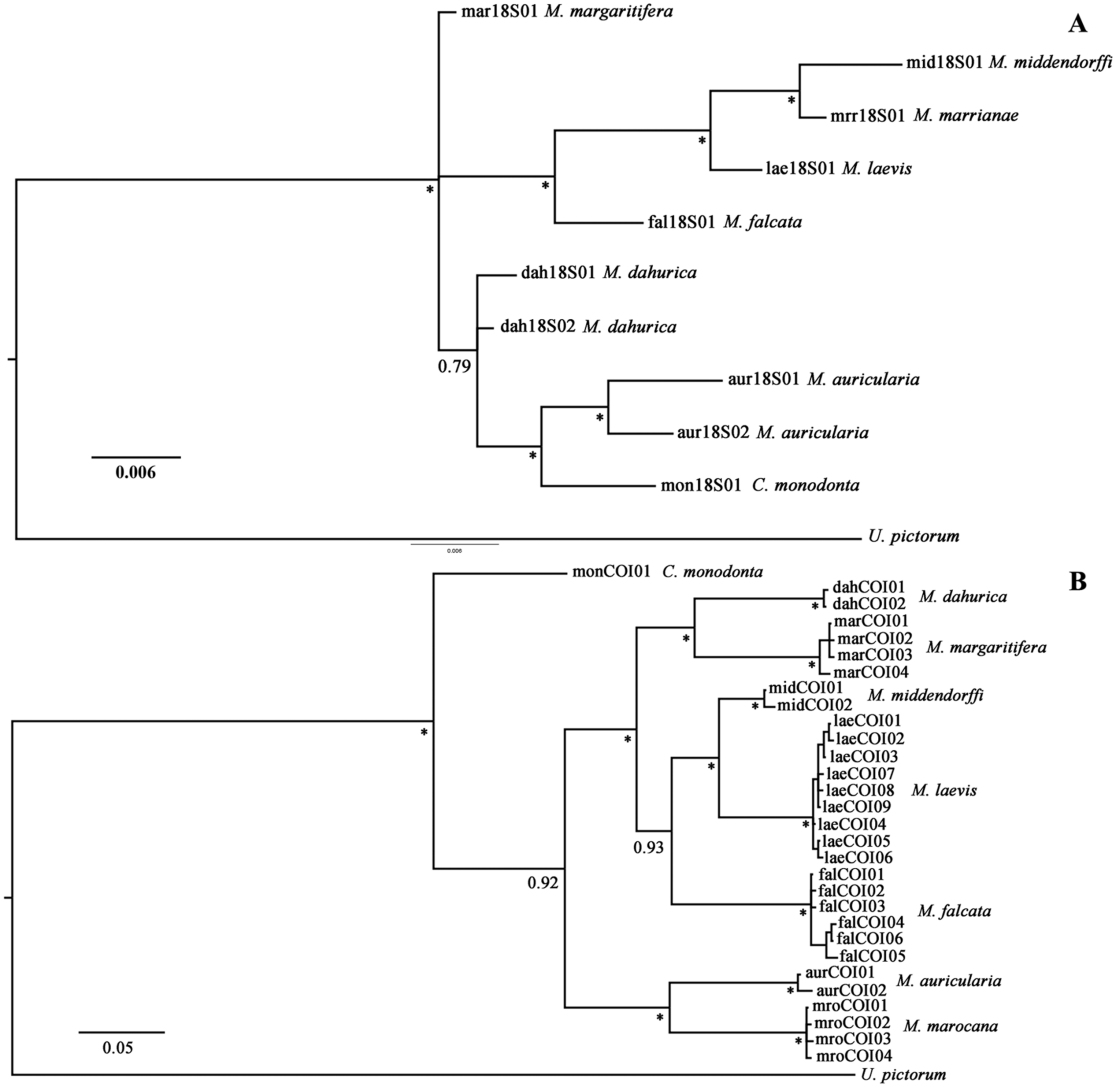
Bayesian phylogeny of *Margaritifera* spp. haplotypes. The scale bar indicates the branch length. Asterisks: Posterior probabilities ≥0.95; other significant support node values are mentioned in the figure. For detailed locality and specimen data for analyzed haplotypes, see the supplementary materials (S1 and S2 Tables). A—The 18S rDNA gene dataset. B—The COI gene dataset.

The Bayesian analysis of the nuclear 18S rRNA gene revealed that the NW Pacific species *M*. *middendorffi* and *M*. *laevis* belong to a well-supported monophyletic clade (BPP 1.00) together with the North American species *M*. *marrianae* and *M*. *falcata* (Fig 2A). Among these species, *M*. *middendorffi* is a sister to *M*. *marrianae* (BPP 1.00). *M*. *dahurica*, *M*. *auricularia* and *C*. *monodonta* cluster together in an unresolved clade (BPP 0.79), but there is a sister relationship between the two latter species (BPP 0.99). *M*. *dahurica* and *M*. *auricularia* both have an intra-specific variation of the nuclear 18S rRNA gene with two close haplotypes in each species.

The analysis of the mitochondrial COI gene showed that *C*. *monodonta* forms a separate clade, which is basal within Margaritiferidae. *M*. *dahurica*, *M*. *margaritifera*, *M*. *middendorffi*, *M*. *laevis* and *M*. *falcata* cluster together in a well-supported clade (BPP 1.00) (Fig 2B). Among these, our Bayesian inference strongly supports the sister relationships within two pairs of species, namely *M*. *dahurica* and *M*. *margaritifera* as the first pair (BPP 1.00), and *M*. *middendorffi* and *M*. *laevis* as the second pair (BPP 0.97). *M*. *falcata* shows a closer affinity to *M*. *middendorffi* and *M*. *laevis* (BPP 0.93). Lastly, the COI phylogeny confirms that *M*. *auricularia* and *M*. *marocana* are sister taxa (BPP 0.98).

### Shell and soft body morphology

The structure of hinge teeth and the shape and relative dimensions of the shell have specific characteristics that are described in detail below in the Taxonomic account of each species. The density of mantle scars has no significant differences (Welch’s test: F = 2.45, df = 16, 38, P = 0.12; (S5 Table)) whereas muscle scar has a high variability among studied taxa.

**Fig 3:**
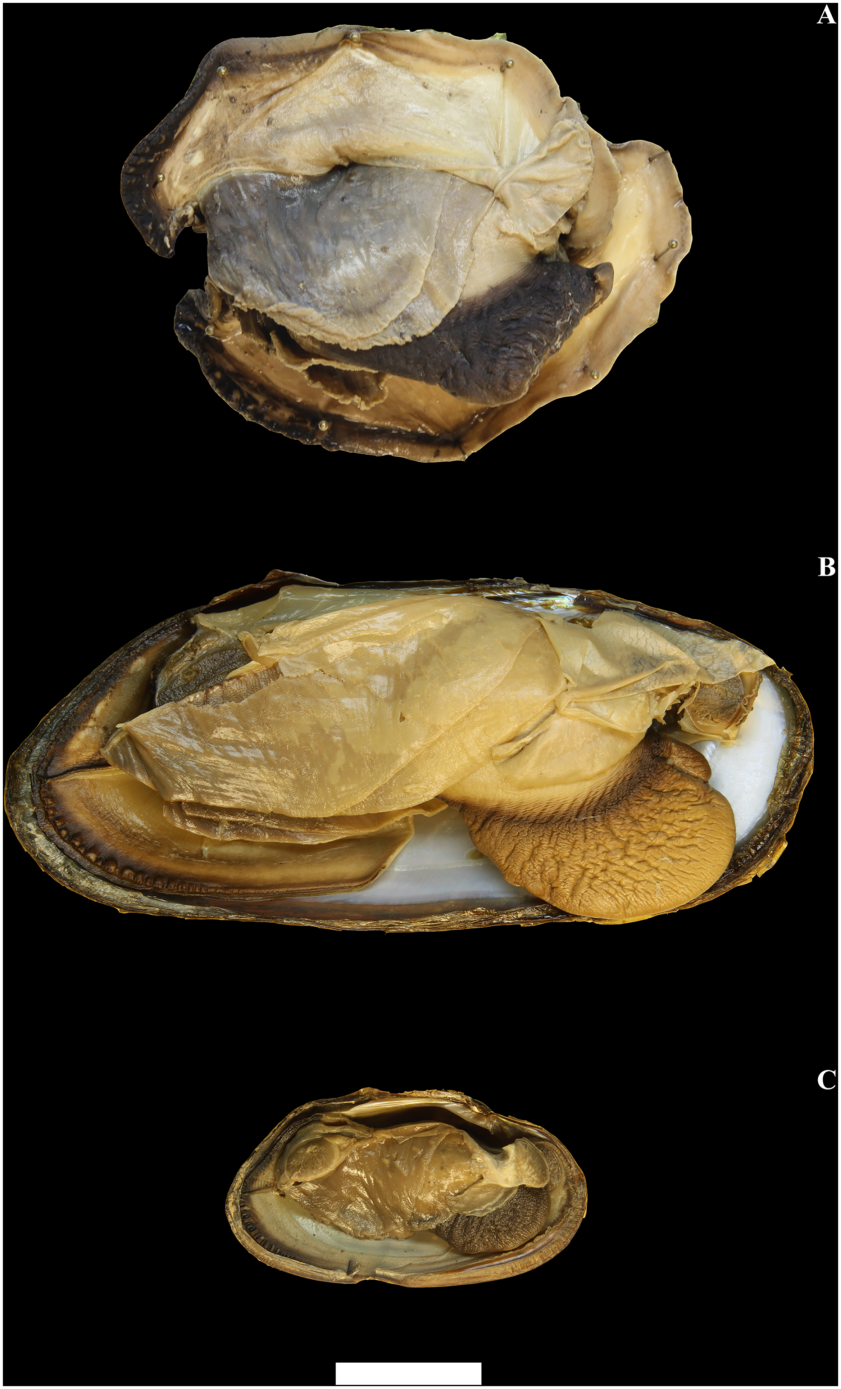
General plan of soft tissue of three Far Eastern margaritiferid species. A—*Margaritifera dahurica* (Middendorff, 1850). B—*M*. *laevis* (Haas, 1910). C—*M*. *middendorffi* (Rosén, 1926). Scale bar—1 cm.

**Fig 4:**
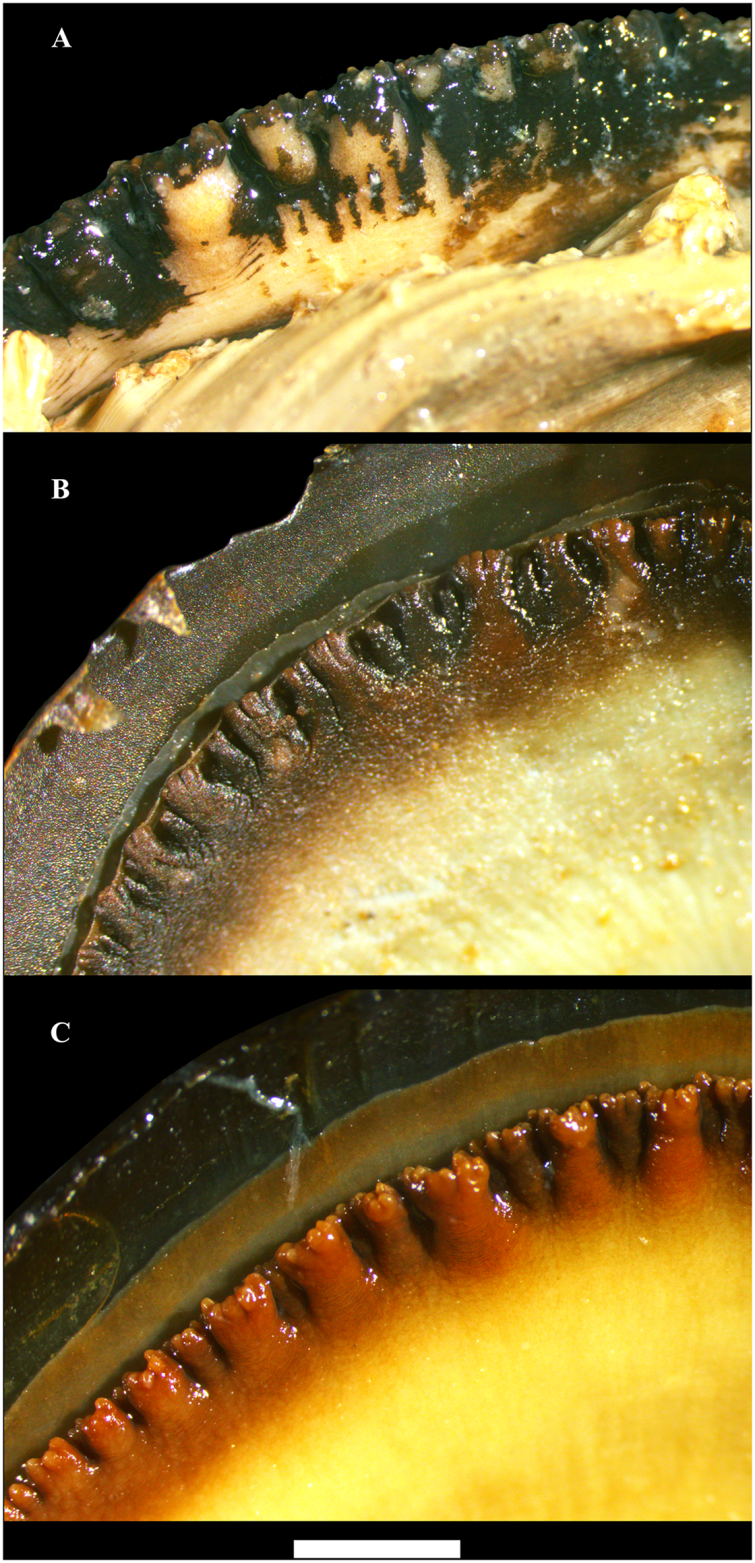
Inhalant siphon morphology of three Far Eastern margaritiferid species. A—*Margaritifera dahurica* (Middendorff, 1850). B—*M*. *middendorffi* (Rosén, 1926). C—*M*. *laevis* (Haas, 1910). Scale bar—2 mm.

A general plan of the whole soft body of *Margaritifera* species is shown in Fig 3. The soft body morphology has more similarity than does the shell morphology among the studied taxa. The mantle color generally is colored cream to cafe au lait with black or brown edges (Fig 3). The gills are cream or light brown. The anterior margin inner gills are slightly longer and wider (higher) than the outer gills (Fig 3). The inner and outer gills are the same size in the middle of the posterior portion. The foot is massive, cream and dark brown distally (Fig 3). The labial palps are half-round, cream to cafe au lait, and convex dorsally. The palps have a smooth external surface and a finely canaliculated inner side. Three apertures are visible in the posterior portion: the supra-anal aperture, the exhalant and the inhalant siphons. This margin of the mantle has dark brown color with a small fold of the exhalant and a large fold of the inhalant siphon (Fig 4). The exhalant and the inhalant siphons are divided by the epithelial fold. The papillae of the inhalant siphon decrease in height in the direction of the ventral margin and are represented by monodactylous outgrowths. The morphological differences between the structures of the inhalant siphon papillae within *Margaritifera* spp. are presented below in the description of the each species.

### Taxonomic account

Family MARGARITIFERIDAE

*Margaritifera* Schumacher, 1816

*Margaritifera dahurica* (Middendorff, 1850)—Amurean freshwater pearl mussel

*Unio* (*Alasmodonta*) *dahuricus* Middendorff, 1850: [43]: 109, [44]: 275–276, pl. 26, Figs 3–5.

*Unio* (*Margaritana*) *dahuricus* Middendorff, 1850: [58]: 699–700.

*Unio margaritiferus* Simpson 1895, partim (ident. err., non Linnaeus, 1758): [59]: 328.

*Margaritana dahurica* (Middendorff, 1850): [23]: 109–112, Fig 35, [24]: 289, Fig 250.

*Margaritifera margaritifera dahurica* (Middendorff, 1850): [60]: 120, [61]: 11.

*Dahurinaia sujfunensis* Moskvicheva, 1973 (comparatory “taxa”): [62]: 1468, Fig 3,e-h.

*Dahurinaia sujfunica* Moskvicheva, 1973 (inadv. err. in the species description). [62]: 1468.

*Dahurinaia tiunovae* Bogatov & Zatravkin, 1988 (comparatory “taxa”): [63]: 156–158, Fig 1, a-b.

*Margaritinopsis dahurica* (Middendorff, 1850): [4]: 42.

*Dahurinaia komarovi* Bogatov et al., 2003 (comparatory “taxa”): [26]: 45–47, Figs 3a & 3d.

*Dahurinaia ussuriensis* Bogatov et al., 2003 (comparatory “taxa”): [26]: 47, Figs 3b & 3g.

*Dahurinaia prozorovae* Bogatov et al., 2003 (comparatory “taxa”): [26]: 48, Figs 3c & 3i.

*Dahurinaia transbaicalica* Klishko, 2008 (comparatory “taxa”): [28]: 292–296, Figs 1–4, 5a.

*Dahurinaia (Kurilinaia) laevis* Klishko, 2009 (ident. err., non Haas, 1910): [64]: 237–238, Figs 1–2.

*Dahurinaia* (*Kurilinaia*) *zatravkini* Klishko, 2009 (ident. err., non Bogatov et al., 2003): [64]: 238–239, Fig 3.

*Margaritifera dahurica* Inoue et al., 2013 (inadv. err.): [65]: Fig 3(a).

**Fig 5:**
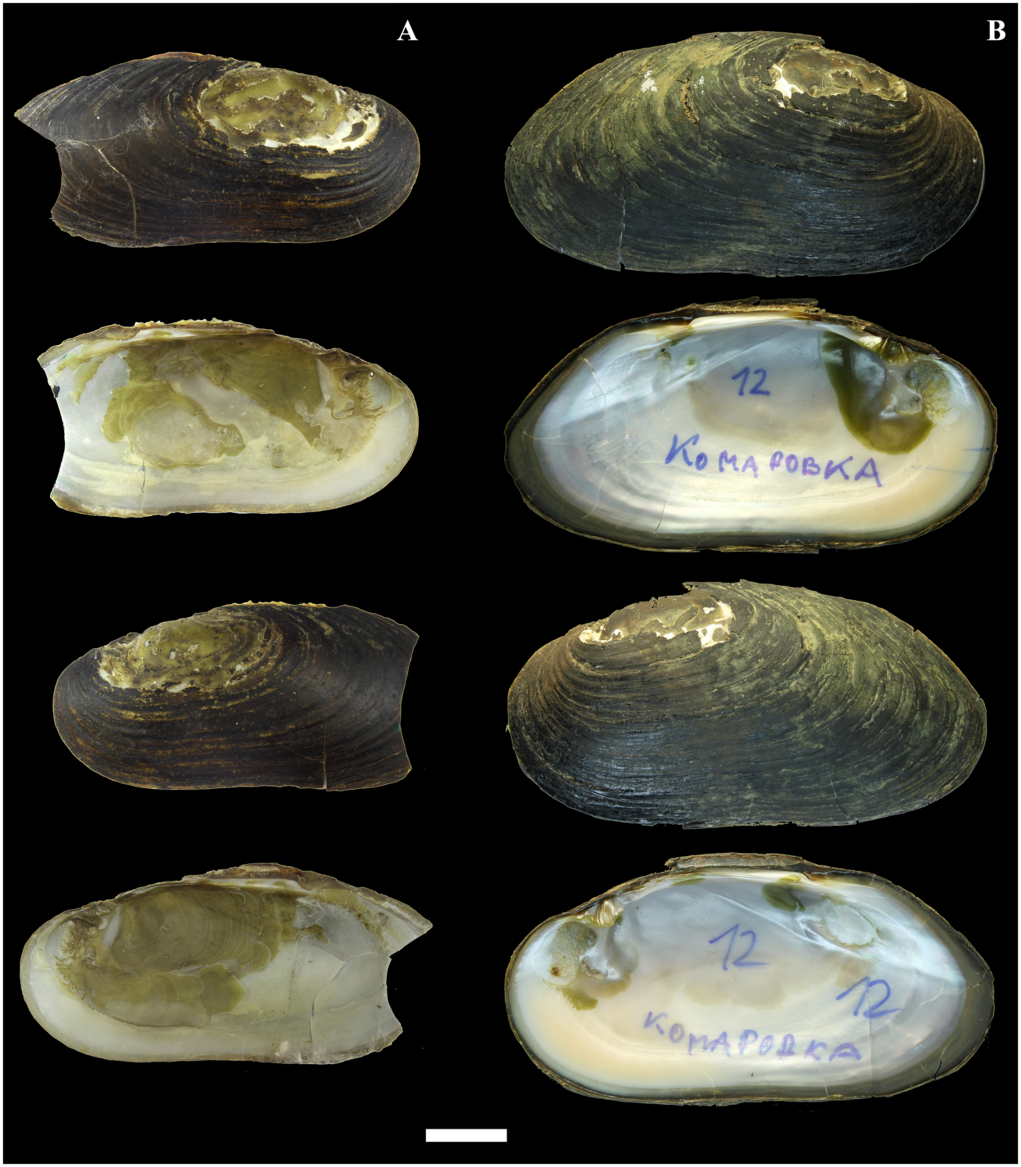
Shells of *Margaritifera dahurica* (Middendorff, 1850). A—Lectotype (ZISP: no. 7a). B—specimen from Komarovka River, Razdolnaya River basin, Primorye. Photos by I.V. Vikhrev. Scale bar—2 cm.

**Fig 6:**
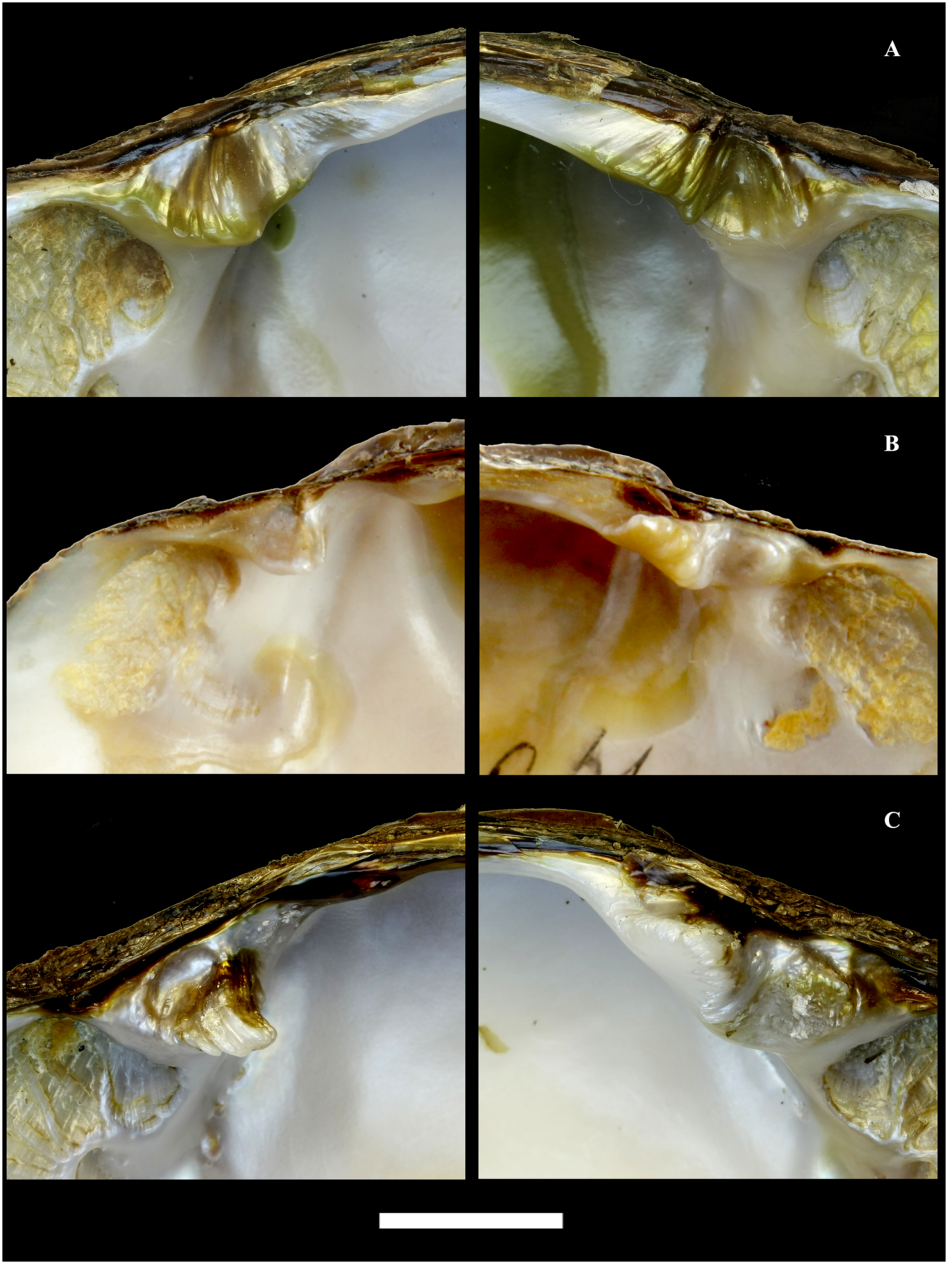
Teeth morphology of three Far Eastern margaritiferid species. A—*Margaritifera dahurica* (Middendorff, 1850). B—*M*. *middendorffi* (Rosén, 1926). C—*M*. *laevis* (Haas, 1910). Scale bar—1 cm.

**Fig 7:**
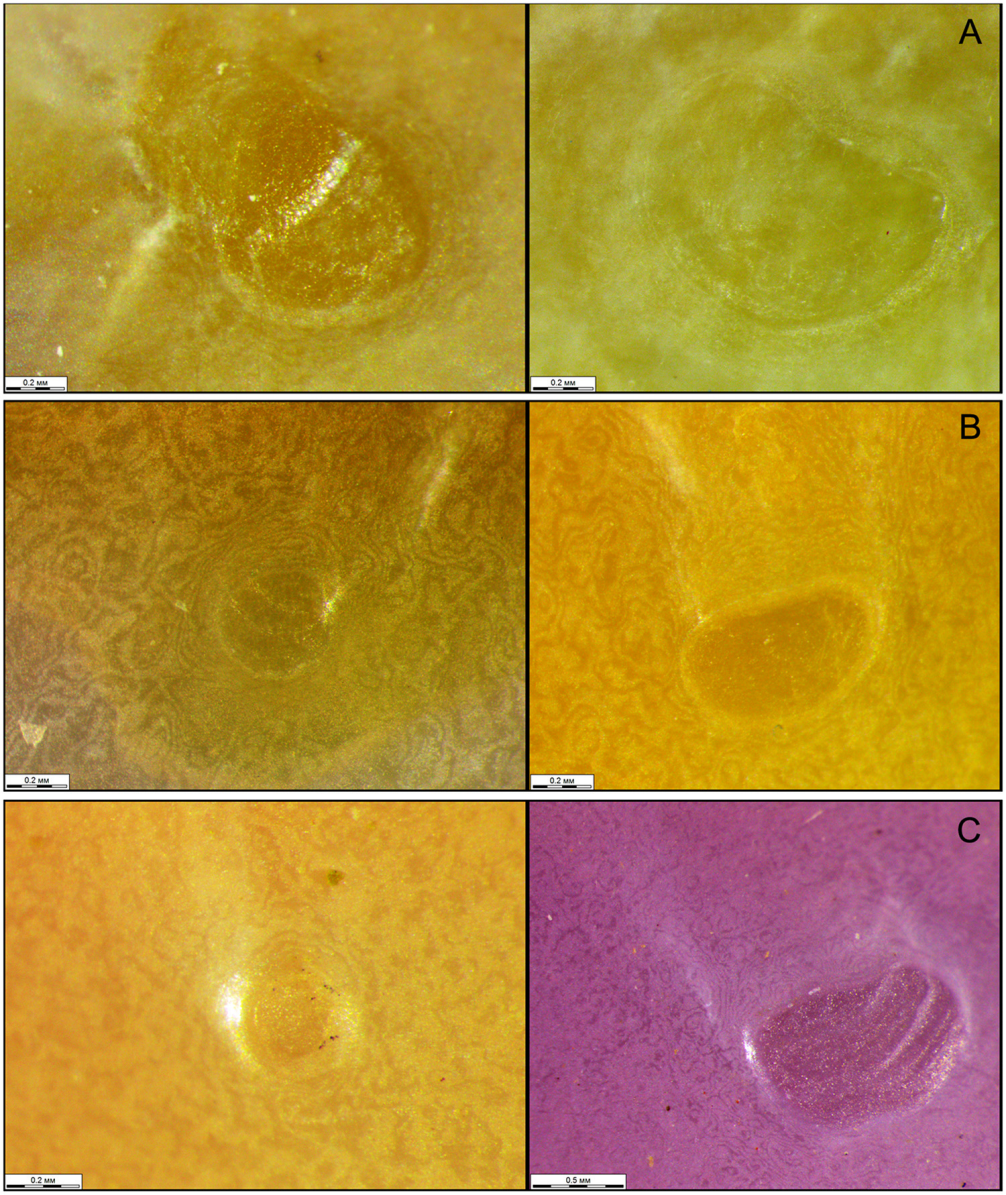
Microphotographs of the mantle attachment scars on shells of four Eastern Asian margaritiferid species. A—*Margaritifera dahurica* (Middendorff, 1850). B—*M*. *middendorffi* (Rosén, 1926). C—*M*. *laevis* (Haas, 1910).

**Fig 8:**
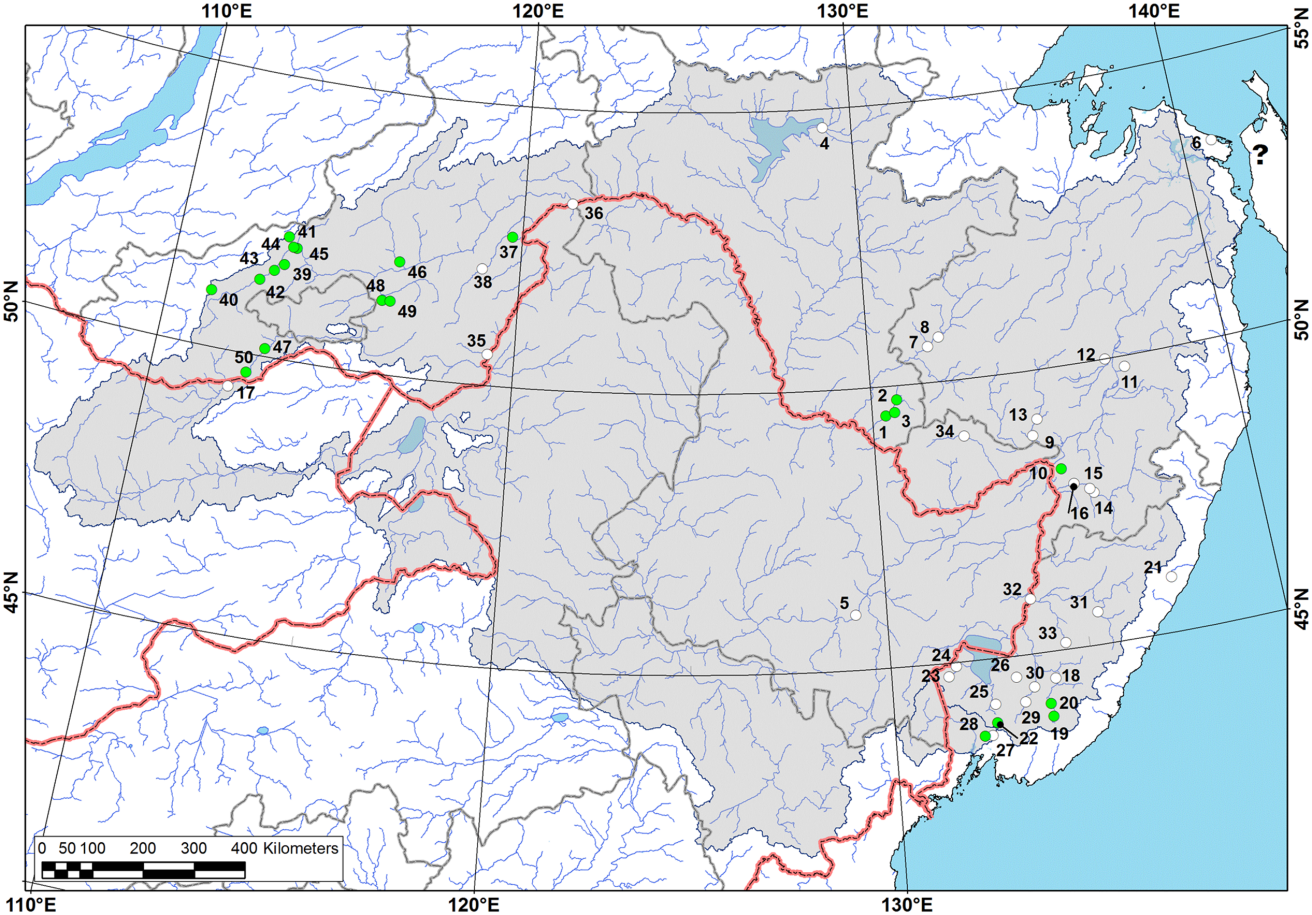
Range map of *Margaritifera dahurica* (Middendorff, 1850). Green circles are representing recent viable populations (observed since 2000), white circles—old records (until 2000). Question mark is indicated an uncertain record from the Langry River [69], [26]. Grey areas are indicated an approximate modern species range (it is shown only for the large river systems). Species locality numbers on the map correspond to numbers in S2 Table.

**Fig 9:**
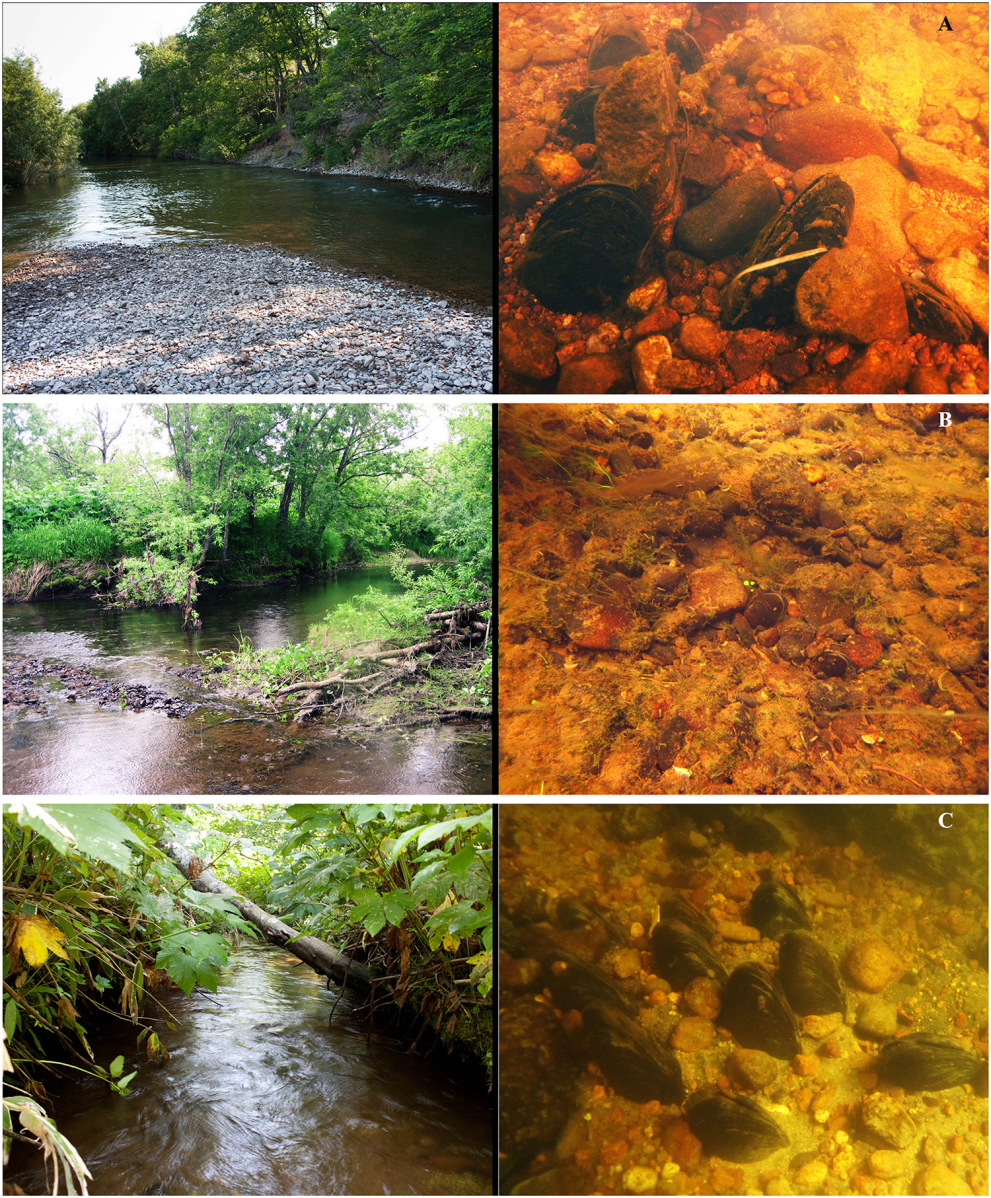
Typical habitats of the Eastern Asian margaritiferid species. A—*Margaritifera dahurica* (Middendorff, 1850): the Ilystaya River, Primorsky Kray. B—*M*. *middendorffi* (Rosén, 1926): the Nachilova River, Kamchatka. C—*M*. *middendorffi* (Rosén, 1926) & *M*. *laevis* (Haas, 1910): the Golovnina River, Kunashir Island. Photos by Y.V. Bespalaya, Y.S. Kolosova & I.V. Vikhrev.

Figs 3A, 4A, 5, 6A, 7A, 8 & 9A.

#### Type locality

“Transbaikalien” [43]: 109; “Am Zusammenflusse des Argunj mit der Schilka” [44]: 276 (confluence of the rivers Argun and Shilka, the Upper Amur basin: see Fig 8 & S2 Table, locality no. 36).

#### Type

The Middendorff’s type series includes two specimens that are deposited in the ZISP collection. Bogatov et al. [26]: 45 designate specimen No. 7a as a lectotype, because it was pictured by Middendorff [44]: pl. 26, Figs 3–5; and assigned the second specimen No. 7b (single valve) as a paralectotype. The lectotype shell has 105 mm length, 32 mm height and 25 mm width. It is noteworthy, that Bogatov et al. [26] were not using the protologue of Middendorff [43] for their revision, and cited only Middendorff’s later work [44].

#### Morphology

The shell is equivalve and inequilateral, large (max length 196.5 mm, N = 221), elongate-oval and flat. The anterior margin is rounded, smoothly following the ventral margin, which is slightly concave in the middle nearer to the ventral edge (Fig 5). The posterior margin is wide, oval and rounded-triangular. The dorsal margin is slightly curved in passing from the posterior margin. The umbos are not projecting. The right and the left valves have only pseudo-cardinal teeth (Fig 6). Lateral teeth are absent or reduced to faintly visible rudimentary lamellae. The right valve has a pseudo-cardinal tooth, which is pyramidal, strong and canted with deep streaks and serrated edge. The anterior pseudo-cardinal tooth of the left valve is inconspicuous, often reduced and similar to a pointed tubercle; the posterior pseudo-cardinal tooth is triangular and slight with unpronounced streaks. The anterior adductor muscle scar is deep and clear; the posterior adductor muscle scar not deep and more weakly pronounced. Mantle attachment scars are deep, rounded and well pronounced and their sharpness varies from rounded to bean-like (Fig 7A). The nacre is white with oil spots. The aperture of the inhalant siphon (Fig 4A) of *M*. *dahurica* has large, tightly fitting papillae. The papillate outgrowths are small and tightly grouped (in the form of inflorescences) on the apex of the papillae. The papillae are branched from the edges of the mantle.

#### Distribution

The Amur Basin (within the territories of Russia, Mongolia and China), the Razdolnaya Basin (upper tributaries), the Peschernaya (Kulumbe) River, the Iska River and the Arey Lake (Fig 8 & S2 Table).

#### Habitats

Different types of watercourses, such as small streams, small and medium sized rivers, as well as large rivers (rivers Ussuri, Arkhara, Shilka, etc.) (Fig 9A). The sole lake population was found in Arey Lake, Transbaikalia, Siberia, Russia [28]; but divers recorded only a few dead shells here in the year 2013 (O. K. Klishko, pers. comm.). Populations prefer sand-gravel and gravel-pebble grounds at the riffles and runs. Sometimes, individual specimens were observed on the clay and silt-sand bottom of the pool river sites.

#### Host fishes

Not known. Actually, a list of seven salmonid species, which can serve as hosts of the *M*. *dahurica* [36], has not been verified experimentally.

#### Remarks

All of the referred comparatory “taxa” of the genus *Dahurinaia* were synonymized with *M*. *dahurica* based on shell morphology [4], [8], [31], [39]. Our molecular data confirmed this decision. For example, Bogatov [29] noted that the single known *Dahurinaia sujfunensis* population inhabits Razdolnaya Basin, which is separate from the Amur drainage. Bogatov reports that “…between the Amur basin and basins of rivers of the south of Primorsky kray, no common species of large bivalves has been found yet, which is explained by the historic development of these basins” [29]: 674. However, according to new molecular and morphological data, the specimens from the Razdolnaya drainage are identical to typical *M*. *dahurica* specimens from different parts of the River Amur Basin.

#### Material examined

See S1 and S2 Tables.

*Margaritifera middendorffi* (Rosén, 1926)—Middendorff’s freshwater pearl mussel

*Unio* (*Alasmodonta*) *complanatus* Middendorff, 1851 (ident. err., non Dillwyn, 1817): [44]: 273–274, pl. 27, Figs 1–6.

*Unio margaritiferus* Simpson 1895, partim (ident. err., non Linnaeus, 1758): [59]: 328.

*Margaritana middendorffi* Rosén, 1926: [46]: 269–270, [23]: 112–114, Fig 36, [24]: 289, Fig 251.

*Margaritana margaritifera middendorffi* Rosén 1926: [61]: 12.

*Dahurinaia middendorffi* (Rosén, 1926): [66]: 16.

*Dauhrinaia middendorffii* Buyanovsky, 1993 (inadv. err.): [67]: 29.

*Margaritinopsis middendorffi* (Rosén, 1926): [4: 42].

*Kurilinaia kamchatica* Bogatov et al., 2003 (comparatory “taxa”): [26]: 48, Figs 4A & 4C.

*Kurilinaia zatravkini* Bogatov et al., 2003, partim (comparatory “taxa”): [26]: 49–50, Figs 4B & 4F.

*Margaritifera laevis* Huff et al., 2004 (ident. err., non Haas, 1910): [38]: 381, GenBank acc. no. AY579124.

**Fig 10:**
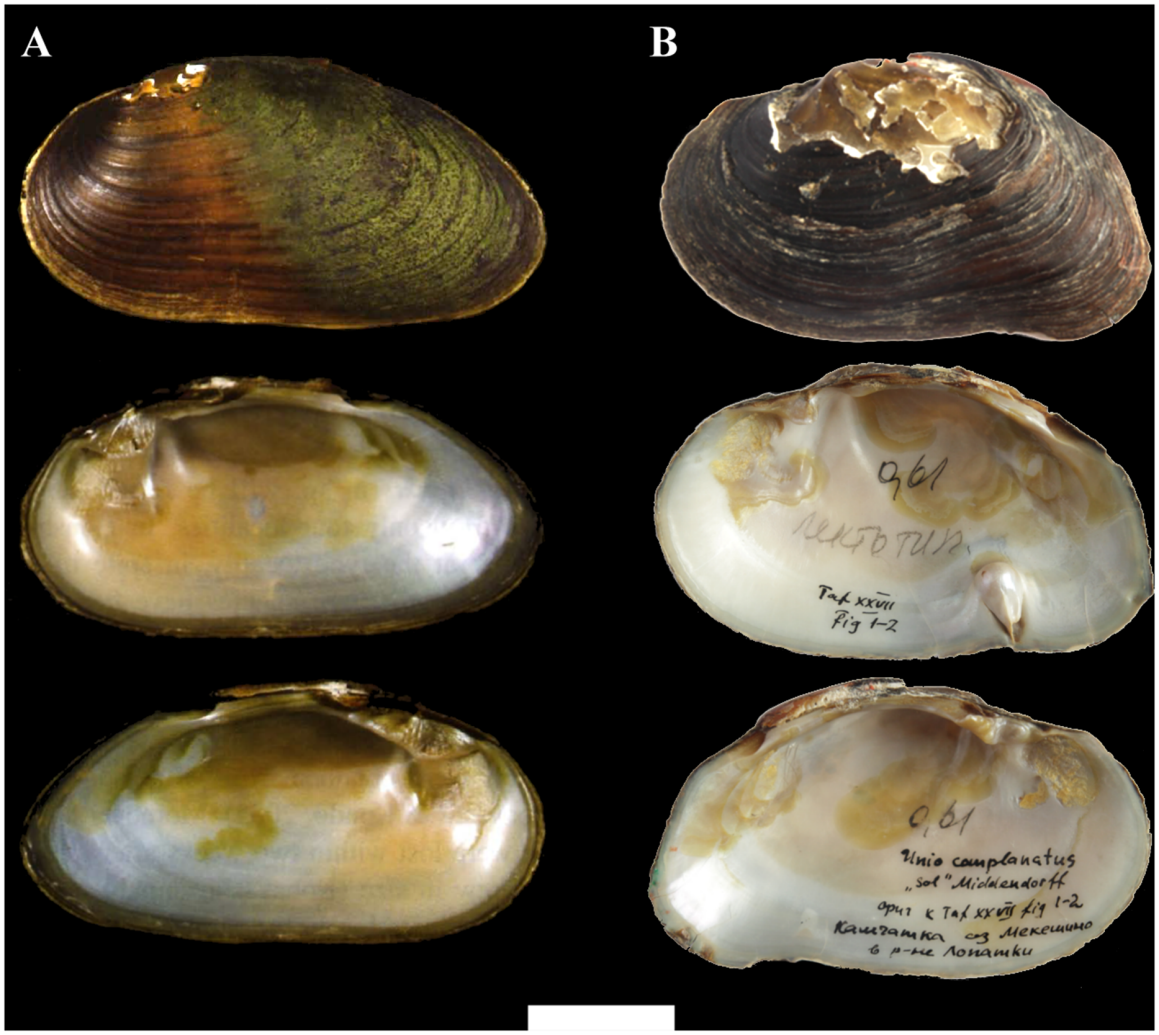
Shells of *Margaritifera togakushiensis* (Kondo and Kobayashi, 2005) and *Margaritifera middendorffi* (Rosén, 1926). A—Holotype of *M*. *togakushiensis* [18]: 137, figs 5–8. B—Lectotype of *M*. *middendorffi* (ZISP: no. 6). Photo by I. V. Vikhrev. Scale bar—2 cm.

**Fig 11:**
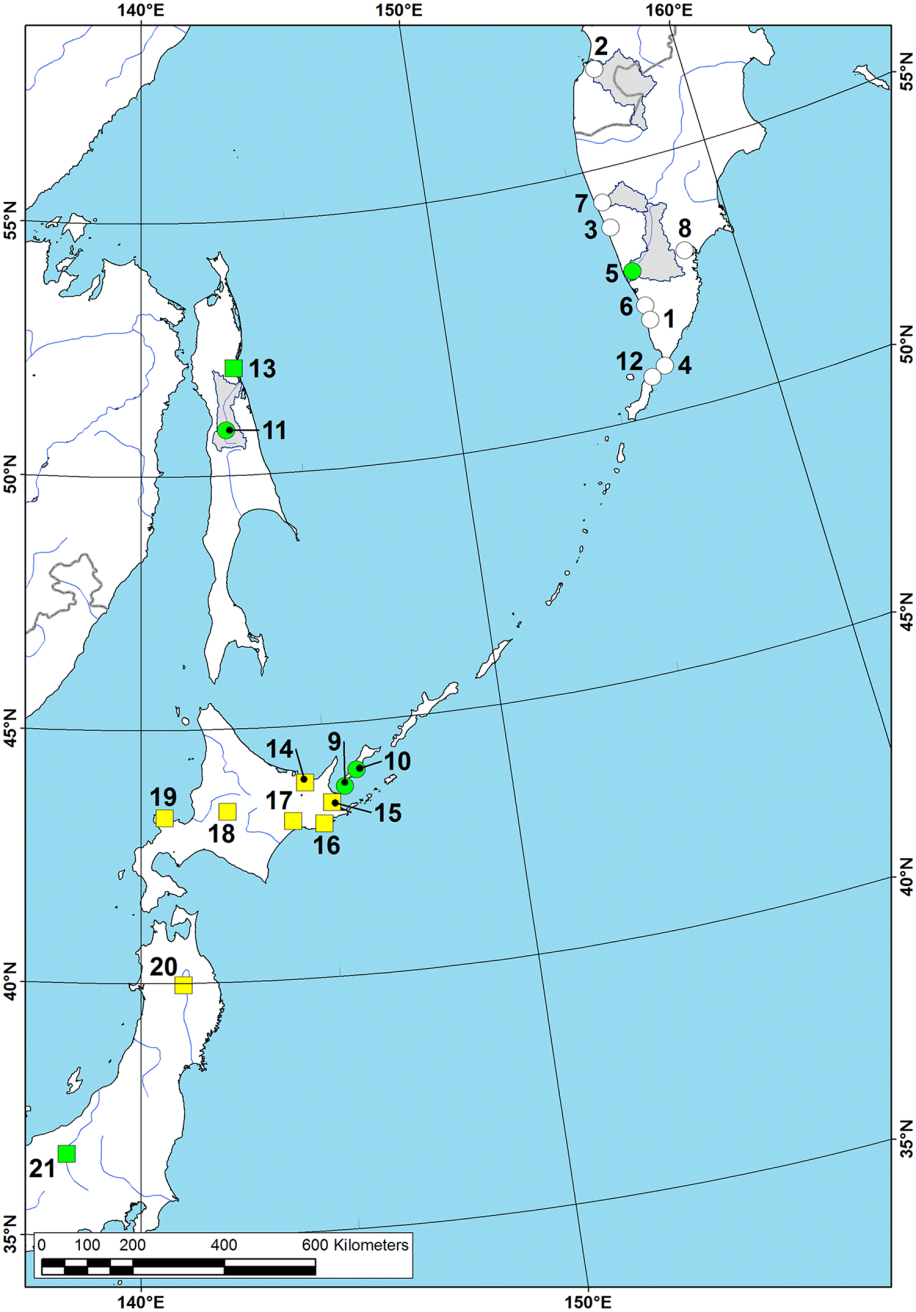
Range map of *Margaritifera middendorffi* (Rosén, 1926) and *Margaritifera togakushiensis* (Kondo and Kobayashi, 2005). Circles—*M*. *middendorffi* locations, squares—*M*. *togakushiensis* locations. Green circles and squares are representing recent viable populations (observed since 2000), white circles—old records (until 2001), yellow squares—records without exact dates. Grey areas indicate approximate modern species ranges (showing only the large river systems). Species locality numbers on the map correspond to numbers in S3 Table.

Figs 3C, 4B, 6B, 7B, 9B, 10 & 11.

#### Type locality

“Reka Golyshka” [the river Golygina, Kamchatka Peninsula: see Fig 11 & S3 Table, locality no. 1] [46]: 269; “Kamtschatka; im See Mjäkéshino des Südendes (Lopatka) dieser Halbinsel” (Kamchatka; the lake Mekeshino on the southernmost extremity of the peninsula: see Fig 11 & S3 Table, locality no. 4) [44]: 274.

#### Type

Middendorff [44]: 273–274, pl. 27, Figs 1–6 described this species as *Unio* (*Alasmodonta*) *complanatus* and pictured two specimens of the species. In his protologue, Rosén [46]: 270 fully noted the Middendorff description and indicated the shell size of one Middendorff specimen as 93 mm in length, 48 mm in height and 32 mm in width. Rosén did not see these specimens but used their images from the work of Middendorff. In addition, Rosén [46] was used for the description of two other specimens that were collected by Mr. Ruvinsky. The larger specimen had a shell length of 79 mm, a shell height of 40 mm and a shell width of 28 mm; the smaller specimen had shell dimensions of 56.25 mm, 33.75 mm and 20 mm, respectively (its left valve had a 3-mm-diameter pear-shaped pearl with a dirty light brown color). Rosén had only this material for the species description, as noted in the paper. Thus, these four specimens constitute a type series of *M*. *middendorffi*; all of these specimens are deposited in the ZISP. Bogatov et al. [26]: 45 designated the specimen as no. 6, which was described and pictured by Middendorff [44]: 273–274, pl. 27, Figs 3–6, as a lectotype. However, Bogatov et al. [26]: 45 erroneously considered 16 specimens as paralectotypes because they included many additional specimens from two type localities into the type series. At the same time, Bogatov et al. [26] replaced both of Rosén’s specimens (true paralectotypes) by a type series of their new comparatory “taxon” *Kurilinaia kamchatica* (one of these specimens was assigned as a holotype of this comparatory “species”). However, we found that only three of these specimens were really paralectotypes of *M*. *middendorffi* (by ZISP catalogue: “paralectotype of *M*. *middendorffi* no. 1”; “holotype of *Kurilinaia kamchatica* no. 1”; “paratype of *K*. *kamchatica* no. 2”).

#### Morphology

The shell is small relative to other margariferid species (max length 93 mm, N = 193), oval, slightly convex and rhomboid in shape (Fig 10). The anterior margin is rounded, smoothly following the slightly curved ventral margin. The posterior margin is angular. The dorsal margin is slightly curved with a prominent umbo. Two pseudo-cardinal teeth and rudiments of two lateral teeth are in the left valve, while a pseudo-cardinal tooth and a rudimentary lateral tooth are in the right valve. The right valve has a thin triangular pseudo-cardinal tooth serrated at the apex. A deep triangular groove passing through a thickened fold is arranged behind the pseudo-cardinal tooth. The anterior pseudo-cardinal tooth in the left valve is short; the posterior pseudo-cardinal tooth is trapezoidal or rarely triangular with deep streaks and is serrated at the apex. The lateral teeth in both valves are presented as thin rudimentary lamellae. The anterior adductor muscle scar is deep; the posterior scar is pronounced more weakly. The mantle attachment scars are not well pronounced (Figs 10 and 7B). The nacre is pink in the anterior edge and bluish-silvery in the posterior edge with oil spots. The papillae of the inhalant siphon are large and differently digitated. The outgrowths on the apex of the papillae of *M*. *middendorffi* are slightly branched in comparison with those of *M*. *laevis* and *M*. *dahurica* (Fig 4B). The papillae are branched below the edges of the mantle.

#### Distribution

Rivers of the Kamchatka Peninsula, Kurile Archipelago, Sakhalin Island (Fig 11 & S3 Table), and likely of Japan.

#### Habitats

Small streams, small- and medium-sized rivers (Fig 9B). Usually, populations were recorded on sand-gravel grounds of the runs but, in the extreme north of the range, the species inhabited only pool river sites. In some rivers of the Sakhalin and Kunashir Islands, Far East of Russia, the species coexists with *M*. *laevis* (see Fig 9B & S3 Table).

#### Host fishes

Not known. Kondo & Kobayashi [18] stated *Salvelinus leucomaenis* as a host fish for *M*. *togakushiensis*. As far as we suppose *M*. *togakushiensis* and *M*. *middendorffi* conspecific, they may have the same host fish. [18].

#### Remarks

Middendorff [44] erroneously identified this species as *Unio complanatus*.

Then, Simpson [59]: 328 noted that Middendorf’s specimens are “…without lateral teeth, and appear to be a stunted form of *Unio margaritiferus*”. Finally, Rosén [46] described *M*. *middendorffi* as a separate species and not as an intra-specific form of *M*. *margaritifera*. However, Haas [61], [60] incorrectly cited Rosén’s name as *Margaritana margaritifera middendorffi*. *Kurilinaia kamchatica* Bogatov et al., 2003 is a comparatory “species” that was recently synonymized with *M*. *middendorffi* [31]. *Kurilinaia zatravkini* Bogatov et al., 2003 is also a comparatory “species”; its holotype belongs to *M*. *laevis*, but among the paratypes, we found four specimens of *M*. *middendorffi* (ZISP: nos. 5, 5a, 7 and 8). Records of *Margaritifera* specimens on Kunashir Island [68]: 134 can pertain to *M*. *middendorffi* as well as to *M*. *laevis*. Therefore, some published references [8], [67], [69], [70] with records from the Sakhalin and Southern Kurile Islands cannot be used without revision of the specimen samples.

#### Material examined

See S1 & S3 Tables.

*Margaritifera laevis* (Haas, 1910)—Japanese freshwater pearl mussel

*Margaritana dahurica* Kobelt, 1879 (ident. err., non Middendorff, 1850): [71]: 427–428, pl. 13, Figs 1–2.

*Unio margaritifer* Schrenck, 1867, partim (ident. err., non Linnaeus, 1758): [58]: 700–704.

*Unio margaritiferus* Simpson, 1895, partim (ident. err., non Linnaeus, 1758): [59]: 303.

*Ptychorhynchus laevis* Haas, 1910: [45]: 498.

*Margaritana sachalinensis* Zhadin, 1938: [23]: 114–115, Fig 37, [24]: 289–291, Fig 252.

*Margaritifera margaritifera laevis* (Haas, 1910): [60]: 120, [61]: 12.

*Dahurinaia kurilensis* Zatravkin & Starobogatov, 1984 (comparatory “taxa”): [72]: 1789–1790, Figs 11–14, [66]: 21.

*Dahurinaia shigini* Zatravkin & Bogatov, 1987: (comparatory “taxa”): [66]: 23–24, Fig 4B.

*Margaritifera* (*Dahurinaia*) *kunahiriensis* Habe, 1991 (inadv. err.): [73]: 3.

*Dauhrinaia kurilensis* Buyanovsky, 1993 (inadv. err.): [67]: 29.

*Margaritana sacchariensis* Bába, 2000 (inadv. err.): [74]: 133.

*Margaritinopsis laevis* (Haas, 1910): [4]: 42.

*Kurilinaia kurilensis* (Zatravkin & Starobogatov): [26]: 42, [27]: 25.

*Kurilinaia laevis* (Haas, 1910): [26]: 45.

*Kurilinaia zatravkini* Bogatov et al., 2003: partim (comparatory “taxa”): [26]: 49–50, Figs 4B & 4F.

**Fig 12:**
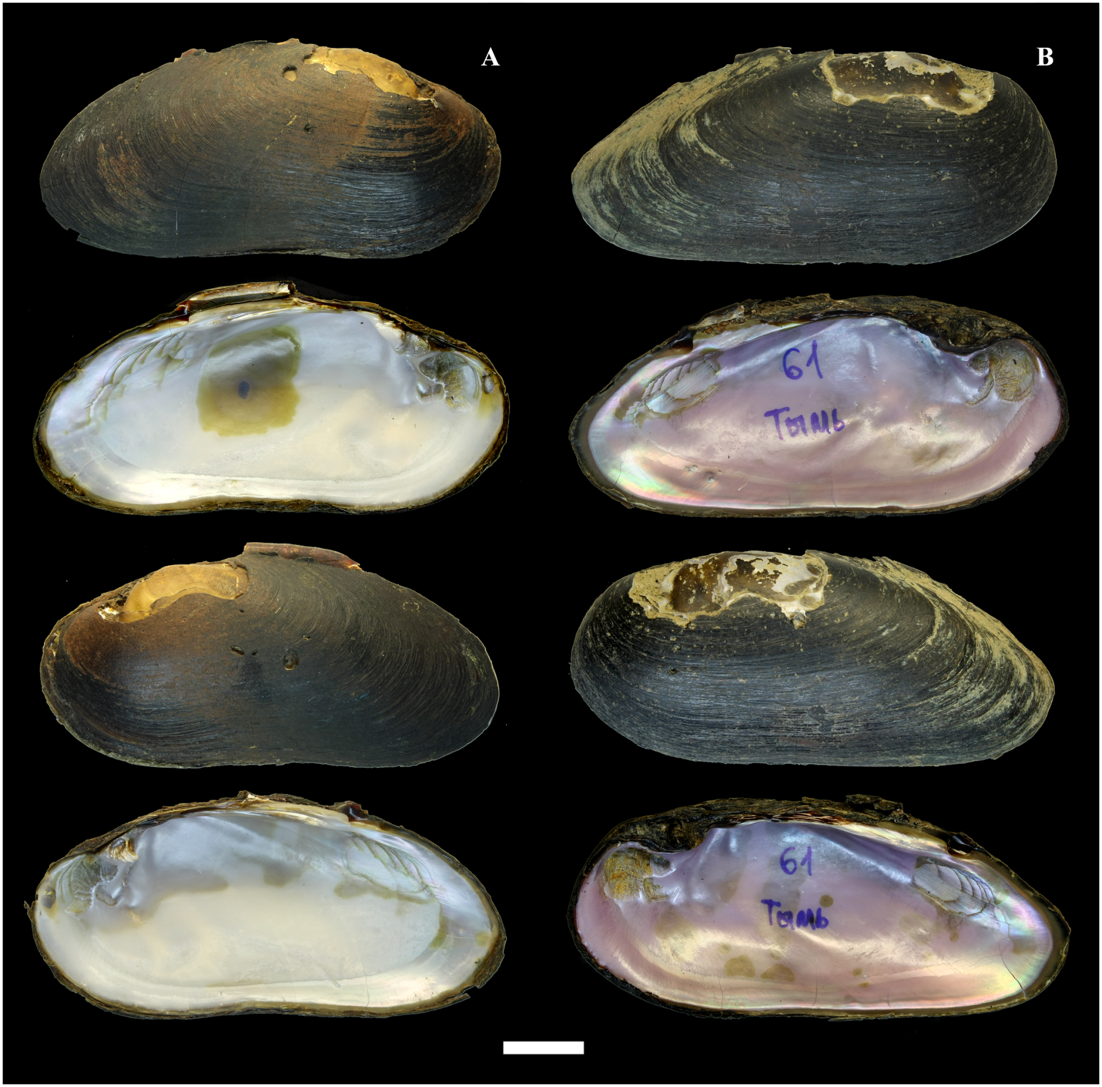
Shells of *Margaritifera laevis* (Haas, 1910). A—Sennaya River, Kunashir Island. B—Tym’ River, Sakhalin Island. Photos by I. V. Vikhrev. Scale bar—2 cm.

**Fig 13:**
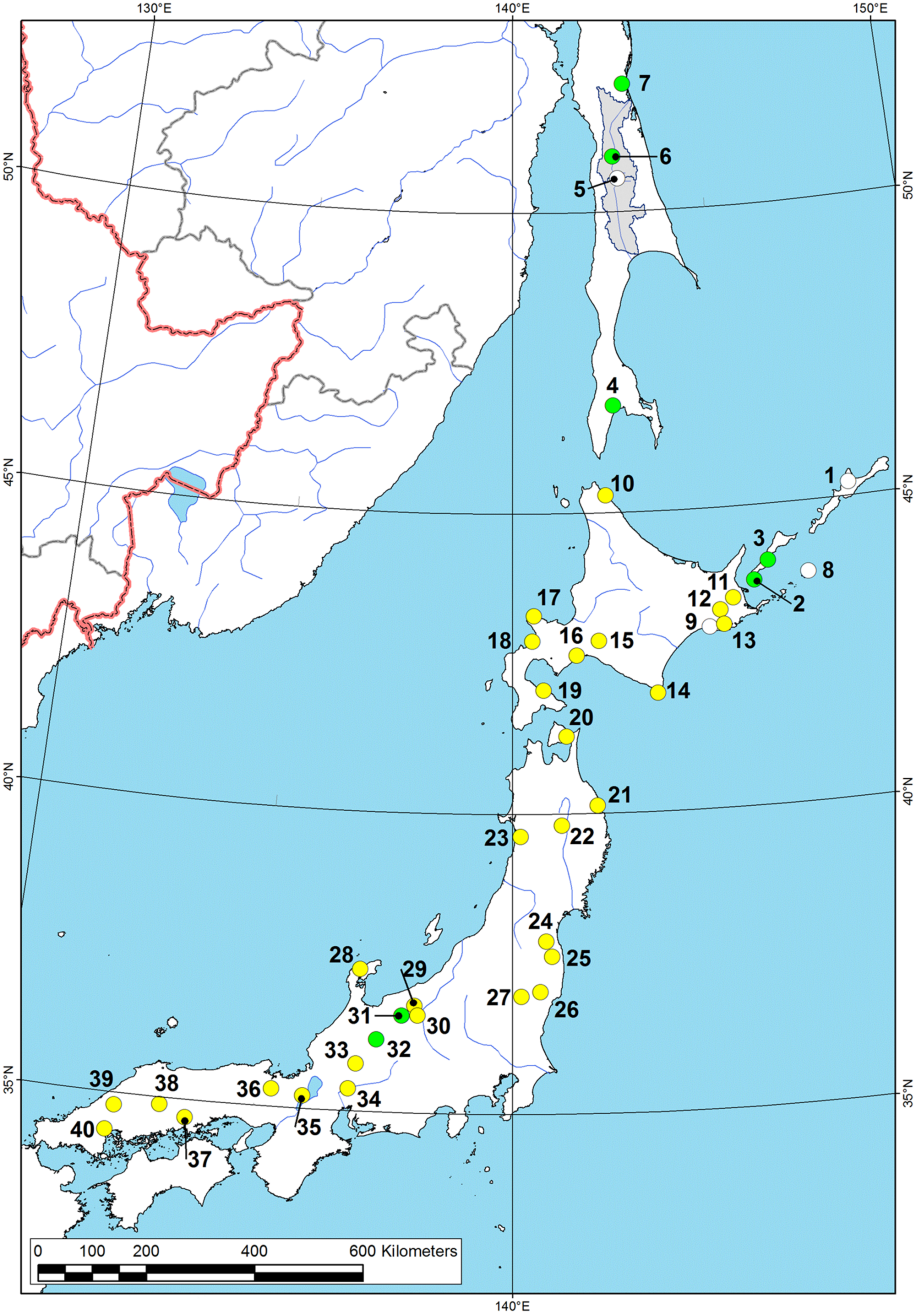
Range map of *Margaritifera laevis* (Haas, 1910). Green circles are representing recent viable populations (observed since 2000), white circles—old records (until 2001), yellow circles—records without exact dates. Grey areas indicate the approximate modern species range (showing only the large river systems). Species locality numbers on the map correspond to numbers in S4 Table.

Figs 3B, 4C, 6C, 7C, 9C, 12 & 13.

#### Type locality

“Sakhalin Island.”

#### Type

Type specimen (holotype) of Haas [45] is deposited in the Senckenberg Museum, Frankfurt (no. SMF3626) [39].

#### Morphology

The shell is large (max length 139.5 mm, N = 140) and elongate-elliptical and sometimes is compressed laterally in large specimens (Fig 12). The anterior margin is rounded; the dorsal margin is slightly curved, almost parallel to the ventral margin and smoothly follows to the posterior margin. The ventral margin is slightly concave or straight. The umbos are not projected. Two pseudo-cardinal teeth and the rudiments of two lateral teeth are in the left valve, while a pseudo-cardinal tooth and a rudimentary lateral tooth are in the right valve. The right valve has a pseudo-cardinal tooth that is strong, high, triangular and serrated. A triangular groove is arranged between the pseudo-cardinal teeth of the left valve. The left valve has an anterior pseudo-cardinal tooth that is triangular, smaller and shorter than the posterior one, which is crenulated from the side groove. The posterior pseudo-cardinal tooth of the left valve is high, pyramidal and serrated peripherally. The lateral teeth in both valves are presented as rudimentary narrow lamellae that are slightly serrated in the distal part. The anterior adductor muscle scar is deep; the posterior scar is unclear. The mantle attachment scars are well pronounced, numerous, sharply rounded with a characteristic groove and arranged diagonally to the shell (Figs 7C and 12). The nacre always has oil spots and is brilliant-violet, white or white in the periphery and pink in the middle of the valve. The inhalant siphon structure of *M*. *laevis* is generally similar to that of *M*. *dahurica* (Fig 4C). However, we noted the following distinctive features: the papillae of the inhalant siphon of *M*. *laevis* are high and branched significantly lower of the mantle edge.

#### Distribution

Sakhalin Island, South Kurile Islands (Kunashir, Iturup and Shikotan), Honshu and Hokkaido Islands (Fig 13). Correspondence on the species occurrence in the Upper Amur Basin, Transbaikalia [64] is based on an erroneous identification; however, all of these specimens belong to *M*. *dahurica* based on morphological and molecular data (See S1 Table).

#### Habitats

Small streams, small- and medium-sized rivers. Usually, populations were recorded on sand-gravel grounds at the run and pool sites. In several rivers of Sakhalin, Kunashir, Honshu and Hokkaido Islands, Islands, the species coexists with *M*. *middendorffi* (see Fig 9C & S4 Table).

#### Host fishes

The masu salmon (*Oncorhynchus masu* (Brevoort, 1856)) [18].

#### Remarks

In the main, species synonymy was provided in previous reviews [4], [18], [31], [39]. *M*. *perdahurica* (Yokoyama, 1932), *M*. *otatumei* (Suzuki, 1942) and *M*. *owadaensis* (Noda, 1970), three fossil Cenozoic margaritiferid species from Japan, were also assumed as *M*. *laevis* representatives [39]. However, the condition of specimens of these species is very poor [75], [76], [77], [78]. It is impossible to reliably compare these fossils with shells of recent margaritiferids. Moreover, most of the known fossil specimens, including *M*. *perdahurica* and *M*. *otatumei*, are ancient and belong to the Eocene or Oligocene by stratigraphic classification [78], [79]. It is unlikely that these fossils belong to recent species, but thorough revision of all specimens and collections of fossil margaritiferids would be necessary, in order to clarify the relationships of these bivalves [80].

#### Material examined

See S1 & S4 Tables.

## Discussion

### How many species are distributed in the rivers of the Russian Far East? What are their detailed recent ranges?

Three *Margaritifera* species inhabit the rivers of the Russian Far East, as discerned from morphological and molecular data: *M*. *dahurica* (Middendorff, 1850), *M*. *middendorffi* (Rosén, 1926) and *M*. *laevis* (Haas, 1910). However, the rivers of Northeastern Russia, where large mainland regions remain unexplored, are still not completely studied; therefore the same species that were previously noted by Smith [4] or additional species, may inhabit the north of the Khabarovsky kray, the Magadan oblast, and the Koryakia and Chukotka districts. These areas are difficult to access and require future studies.

*M*. *dahurica* has the largest range among the Eastern Asian species in the genus (see Fig 8). This species is found in almost all of the major tributaries of Amur Basin, an area of approximately two million km^2^, as well as in Razdolnaya Basin and in two separate small rivers. A similar distribution pattern was found in some freshwater fish species, among which approximately 16 were endemic to this area [81]. We found only a single old record of *M*. *dahurica* from Sungari River, the largest Chinese tributary of the Amur, and a few occurrences from Mongolia. However, this species is likely widely distributed in part of the Amur River system within the territory of those countries. Dashi-Dorgi [82] reported *M*. *dahurica* as one of the most common mussel species in the rivers of Eastern Mongolia. This species is not found in the rivers of the Kurile Islands and the most of Sakhalin Island. Bogatov [69] and Bogatov et al. [26] reported a few specimens from the Langry River, which is situated on the extreme northwest of the Sakhalin Island. It is possible that because this river empties into the Amur Estuary, this river might have belonged to the ancient system of the Paleo-Amur [81]. The range of some Amurean fish species also includes rivers in northwestern Sakhalin Island [83]. Nevertheless, the species identification is in need of revision.

*M*. *middendorffi* is also a widespread species that inhabits many rivers across the Northwestern Pacific, from Kunashir Island to the Kamchatka (see Fig 11). Previously, this taxon was considered a local endemic for Kamchatka [4], [23], [24], [25], [31]. It is interesting that *M*. *middendorffi* ranges across the Bussol Strait, lying between the Urup and Simushir Islands (Central Kurile), which is the most significant biogeographical boundary within the Kurile Archipelago [84]. *M*. *middendorffi* and *M*. *togakushiensis* seem to be conspecific because of similar morphological patterns, small shell size (<100 mm) and overlapped ranges. However, the complicated relationships between these poorly known Far Eastern *Margaritifera* taxa preclude final taxonomic solution, which must be carefully verified using sequences for specimens from the type locality of *M*. *togakushiensis* (type series or newly collected topotypes).

The number of *M*. *middendorffi* localities increases northward. For example, on Kunashir Island, only three populations were found, but this species successfully inhabits the cold subarctic rivers of the Kamchatka and Northern Kuriles. Using data from the literature, this species ranges only in the rivers of the Western Kamchatka, Okhotsk Sea Basin [4], [8], [25]. This phenomenon is explained by the fact that these rivers in the Pleistocene were tributaries of the Paleo-Penzhina River system [25]. Therefore, the report by Eyerdam [85] of an occurrence of *Margaritifera* shell in the downstream part of the river Kamchatka (Pacific drainage) was questioned [25]. However, we found a reliable sample of shells from the Paratunka River of Eastern Kamchatka, which flows into the Pacific Ocean (see S3 Table, loc. no. 8). These data indicate that the *Margaritifera* record from the Kamchatka River is most likely true and that this species has a wider range on the Kamchatka Peninsula, but a large part of the region is difficult to access and is still poorly investigated.

From our data, the range of *M*. *laevis* is significantly narrower than the distribution of the previous species (see Fig 13). This species has a more southerly distribution, and has not been found north of central Sakhalin. However, the host fish *Oncorhynchus masu* is much more widespread northward up to Kamchatka Peninsula. Most of the known localities of *M*. *laevis* are situated on the Honshu Island, and their number is linearly reduced to the north. A few fish species have a distribution resembling this, including the Sakhalin Taimen (*Hucho perryi* (Brevoort, 1856)) [83].

### What are the phylogenetic relationships of the Margaritiferidae from the Russian Far East?

Based on the nuclear 18S rRNA gene, *M*. *laevis* and *M*. *middendorffi* cluster together with two North American species, *M*. *falcata* and *M*. *marrianae*. These results support the hypothesis of an ancient Beringian exchange between the freshwater mussel faunas of Northeastern Asia and North America [86]. These data also correspond to results of molecular studies of cyprinid fishes of Eurasia and North America, where the Far Eastern phoxinins have dispersed from North America to the Far East across the Beringia land bridge during the Late Cretaceous or Early Paleocene [87]. It is interesting, that recent populations of *M*. *marrianae* are isolated in a few tributaries of the Alabama and the Conecuh (Escambia) rivers [4], [88]. It is known that North American Margaritiferidae are not limited strictly to drainages of the Great Basin and Pacific Coast but also occur in parts of the upper Missouri drainage, where they are represented by one species *M*. *falcata* [86].

According to the COI gene sequences, we found a similar “Beringian” clade, formed of *M*. *laevis*, *M*. *middendorffi* and *M*. *falcata*. Unfortunately, the position of *M*. *marrianae* based on the COI phylogeny remains uncertain, because there are only three very short COI sequences for this species in NCBI’s Genbank, which are unsuitable for phylogenetic assessment. However, the minimal COI distances were found between *M*. *laevis* and two other species, *M*. *marrianae* (4.7%) and *M*. *middendorffi* (5.5%), which is in agreement with results, obtained from 18S sequences. *M*. *dahurica* is the sister species of *M*. *margaritifera*, which is distributed across Western Europe and in the river basins of the Atlantic coast of North America [4], [8], [10], [38], [65]. Similar patterns have been found for several freshwater fishes that have closely related representatives from Amur Basin and European rivers, with a huge gap in the Siberian Plain [89], [90], [91], [92], [93]. Most Eastern Asian species have very low intraspecific COI divergence, particularly *M*. *dahurica* and *M*. *middendorffi*. For each of these species, only two COI haplotypes were observed. For twelve examined *M*. *margaritifera* populations, a lack of differentiation was observed in the COI and 16S genes, and these populations may be considered as a metapopulation [9]. A survey of five populations of North American species *Margaritifera hembeli* (Conrad, 1838) showed extensive allozyme monomorphism [94]. These authors mentioned that low genetic diversity appears to be characteristic of Margaritiferidae, as an ANOVA indicated that mussels of the family Margaritiferidae (only *M*. *auricularia*, *M*. *margaritifera* and *M*. *hembeli* were tested) have a significantly lower heterozygosity level than do mussels of the family Unionidae. These authors assumed that, although bottlenecks are known to cause low genetic variability, Margaritiferidae might exhibit metapopulation structure with extinction/re-colonization dynamics leading to low genetic variability. Meanwhile, both COI and microsatellite loci have substantially high levels of genetic variation between *C*. *monodonta* populations that are, most likely, associated with the Pleistocene history of the Mississippi Basin [65]. However, the genetic diversity within each of the populations was low, indicating high gene flow after isolation during the last glacial period.

### Is it possible to reliably identify the Far Eastern species using morphological patterns?

In this study, we analyzed the applicability of specific shell features that were indicated in previous studies for reliable species identification. Three pearl mussel species were identified using a complex of morphology patterns and molecular data. The development and expression of hinge teeth, the shape and the relative dimensions of the shell, the mantle attachment scars, and the muscle scar shape are the most used conchological key features [4], [10], [18], [23], [24], [34], [38], [47], [43], [44], [45], [46], [95], [96], [97], [98], [99].

The dimensions of the shell are the least reliable morphological traits in Unionoida, upon which to base a description [4], [32] due to high variability and the dependence on environmental gradients [4], [33], [34], [100], [101]. However, sometimes, the shell dimensions and shape are useful for distinguishing several Margaritiferidae species. For example, *M*. *middendorffi* has a small shell length (<100 mm) (Fig 10), and *M*. *dahurica* has the largest shell length (up to 196.5 mm) among the Far Eastern pearl mussels (Fig 5).

According to Kondo & Kobayashi [18], the adductor muscle scar shape is one of the key features for *M*. *togakushiensis* identification. However, according to our data, this feature has a high variability and cannot be used for species identification among studied taxa. In the review of recent Margaritiferidae by Smith [4], this feature was also not mentioned as a diagnostic. The adductor muscle scar shape was not used in the morphological descriptions of other unionoid species [10], [86], [95], [96], [97].

The structure and expression of pseudo-cardinal and lateral teeth have a low interspecific variability (Fig 6) and could be reliable characteristics for identification of Margaritiferidae species [4]. The specific characteristics of the structure of the hinge plate in the right valve of *M*. *middendorffi*, namely the arranged deep groove passing through a thickened fold, are typical only for this species and are present in adults, as well as juvenile individuals. Zhadin [23] indicated this key feature, but it was not mentioned within hinge descriptions in later studies of the species [86]. The reduced anterior pseudo-cardinal tooth of the left valve is the basic distinctive feature for *M*. *dahurica* that has been noted in numerous studies [4], [23], [24], [28], [43], [98]. The lateral teeth of *M*. *laevis* are present as thin rudimental lamellae in both of the valves [4], [18], [23], [24]. In all other species of the Far Eastern pearl mussels, lateral teeth are absent or are present as weakly pronounced rudimentary lamellae.

According to Smith [4], [99], the mantle attachment scars on the inner surface of the valves are formed by modified epithelial cells, and specific patterns of the distribution and density of the scars can be identified among taxa. Therefore, the presence of mantle-attachment scars in *M*. *marocana* was sufficient to separate *M*. *marocana* and *M*. *auricularia* [10]. However, our data do not confirm significance of this character for species identification within Margaritiferidae. Fig 7 shows that the mantle scar shape has a high intra-specific variability that overlaps with the differences between taxa. Based on visual assessments, it seems that *M*. *middendorffi* has weaker and less frequent scars than *M*. *dahurica* or *M*. *laevis*.

In addition to shell morphology, the soft body details were also analyzed. The structure of inhalant siphon papillae is often used for species identification [28], [34], [95], [96], [97]. The Far Eastern pearl mussels have differences in this organ. The length of papillae, their frequency, the characteristics of papillae outgrowth, and the position with respect to the mantle edge can be patterns for species identification. Our data confirm the previous description of the *M*. *dahurica* inhalant siphon [28], [34], whose papillae are large and have variable widths. The papillae in the siphon of *M*. *dahurica* are branched over the mantle edges, in contrast to the papillae of *M*. *laevis* and *M*. *middendorffi*. In addition, the papillae of *M*. *laevis* are more branched but in other respects, their structure is similar to that of the papillae of *M*. *dahurica*. Similarly to the mantle attachment scar pattern, the structure of the inhalant siphon of *M*. *middendorffi* is the most distinct; its papillae are less branched and thinner. The structure of the gills and labial palps have no specific features and, despite some differences, could not be used to identify *Margaritifera* spp.

## Conclusions

The present study focuses on the freshwater pearl mussels of the genus *Margaritifera*. This study provides novel insight into the taxonomy of the Far Eastern Margaritiferidae species, their re-description based on morphological patterns, the molecular identification of each species and reliable data for the species ranges, including maps and locality information with precise coordinates. Our results provide an important framework for further research on the phylogeny, ecology, life history and biogeography of one of the most stenobiontic freshwater faunas. Future studies should focus on the unexplored areas of Eastern Asia, including the Russian Far East (the northern region of the Khabarovsky kray, the Magadan oblast, and the Koryakia and Chukotka districts), Korea and Eastern China. Currently, we have negligible information on the freshwater bivalves of these huge regions, which is one of the largest gaps in the current biogeography of the freshwater pearl mussels. Thus, further research should form a basis for understanding the evolutionary history and biogeographical patterns of these freshwater “living fossils” of ancient Laurasian origin.

## Supporting Information

S1 Table List of sequenced *Margaritifera* specimens including species, localities and voucher details as well as NCBI GenBank accession numbers. (DOC)

S2 Table List of known localities of *Margaritifera dahurica* (Middendorff, 1850). (DOC)

S3 Table List of known localities of *Margaritifera middendorffi* (Rosén, 1926) and *Margaritifera togakushiensis* (Kondo and Kobayashi, 2005). (DOC)

S4 Table List of known localities of *Margaritifera laevis* (Haas, 1910). (DOC)

S5 Table Variance and significance tests results for mantle attachment scars density within three margaritiferid species. (DOC)

S6 Table Additional species sequences that were used in the analyses with NCBI Genbank accession numbers. (DOC)
