# Supplementary material for: Taxonomy and Distribution of Freshwater Pearl Mussels (Unionoida: Margaritiferidae) of the Russian Far East
Source: PLoS One. 2015 May 26;10(5):e0122408. doi: 10.1371/journal.pone.0122408 (PMC4444039; doi:10.1371/journal.pone.0122408)
Supplement: S1 Table — (DOC) [file pone.0122408.s001.doc]

**Table S1**. List of sequenced specimens include species status, locality and voucher details as well as GenBank accession numbers

| Species | Locality | Coordinates of locality | DNA voucher no. | Specimen  voucher no. | *COI* | *COI*  haplotype | *18S* | *18s* haplotype |
| --- | --- | --- | --- | --- | --- | --- | --- | --- |
| *M. dahurica* | Komarovka River (Razdolnaya drainage), Primorsky kray, Russia | 43°38’21” N,  132°09’41” E | 99-2  99-9  99-12 | IEPN d0089/2  IEPN d0089/9  IEPN d0089/12 | KJ161518  KJ161519  KJ161520 | dahCOI-1  dahCOI-2  dahCOI-2 | KJ943530  --  KJ943531 | dah18-2  --  dah18-1 |
|  | Ussuri River (Amur drainage), Primorsky kray, Russia | 44°04’52” N,  133°50’43” E | 96-3  96-17  97-7 | IEPN d0093/3  IEPN d0093/17  IEPN d0094/7 | KJ161521  KJ161522  KJ161523 | dahCOI-1  dahCOI-1  dahCOI-1 | --  --  KJ943532 | --  --  dah18-1 |
|  | Ilistaya River (Amur drainage), Primorsky kray, Russia | 43°51’16” N,  132°29’50” E | 92-4  92-6  92-28 | IEPN d0088/4  IEPN d0088/6  IEPN d0088/28 | KJ161515  KJ161516  KJ161517 | dahCOI-1  dahCOI-1  dahCOI-1 | --  KJ943529  -- | --  dah18-1  -- |
|  | Ingoda River (Amur drainage), Zabaikalsky kray, Russia | 51°59’29” N,  113°26’54” E | 71  74  85 | INREC 0052(1)/71  INREC 0052(1)/74  INREC 0052(1)/85 | KJ161525  KJ161526  KJ161530 | dahCOI-1  dahCOI-1  dahCOI-1 | KJ943533  --  KJ943534 | dah18-1  --  dah18-1 |
|  | Onon River (Amur drainage), Zabaikalsky kray, Russia | 49°40’01”N,  112°38’14”E | 70  78  80 | INREC 0052(2)/70  INREC 0052(2)/78  INREC 0052(2)/80 | KJ161524  KJ161527  KJ161528 | dahCOI-1  dahCOI-1  dahCOI-2 | --  KJ943535  -- | --  dah18-1  -- |
|  | Nercha River (Amur drainage), Zabaikalsky kray, Russia | 52°01’34” N,  116°28’32” E | 83 | INREC 0052(2)/83 | KJ161529 | dahCOI-2 | -- | -- |
| *M. middendorffi* | Nachilova River, Kamchatka Peninsula, Russia | 52°53’06” N,  156°26’54” E | 93-1  93-6  93-8 | IEPN d0099/1  IEPN d0099/6  IEPN d0099/8 | KJ161546  KJ161547  KJ161548 | midCOI-1  midCOI-1  midCOI-1 | KJ943539  KJ943540  KJ943541 | mid18-1  mid18-1  mid18-1 |
|  | Tym’ River, Sakhalin Island, Russia | 50°54’45” N,  142°39’48” E | 94-68  94-81  94-86 | IEPN d0082/68  IEPN d0082/81  IEPN d0082/86 | KJ161543  KJ161544  KJ161545 | midCOI-1  midCOI-1  midCOI-1 | --  KJ943538  -- | --  mid18-1  -- |
|  | Confluence of Adamka and Armudanka rivers (Tym’ drainage), Sakhalin Island, Russia | 50°49’42” N,  142°33’18” E | 95-4  95-7  95-15 | IEPN d0079/4  IEPN d0079/7  IEPN d0079/15 | KJ161540  KJ161541  KJ161542 | midCOI-1  midCOI-1  midCOI-1 | KJ943537  --  -- | mid18-1  --  -- |
|  | Voskresenovka River (Tym’ drainage), Sakhalin Island, Russia | 50°54’10” N,  142°39’04” E | 100-1  100-3  100-5 | IEPN e0001/1  IEPN e0001/3  IEPN e0001/5 | KJ161549  KJ161550  KJ161551 | midCOI-1  midCOI-1  midCOI-1 | --  --  -- | --  --  -- |
|  | Golovnina River, Kunashir Island, Kurile Islands, Russia | 43°44’57” N,  145°30’25” E | 264-14  264-16 | IEPN d0044/14  IEPN d0044/16 | KJ161538  KJ161539 | midCOI-1  midCOI-1 | KJ943536  -- | mid18-1  -- |
|  | Serebryanka River, Kunashir Island, Kurile Islands, Russia | 44°03’18” N,  145°51’15” E | 263-18  263-20 | IEPN d0022/18  IEPN d0022/20 | KJ161534  KJ161535 | midCOI-1  midCOI-1 | --  -- | --  -- |
|  | Sennaya River, Kunashir Island, Kurile Islands, Russia | 43°44’56” N,  145°27’57” E | 259-25  259-26 | IEPN d0036/25  IEPN d0036/26 | KJ161536  KJ161537 | midCOI-1  midCOI-1 | --  -- | --  -- |
| *M. laevis* | Tym’ River, Sakhalin Island, Russia | 50°54’45” N,  142°39’48” E | 94-57  94-60  94-83 | IEPN d0082/57  IEPN d0082/60  IEPN d0082/83 | KJ161512  KJ161513  KJ161514 | laevCOI-3  laevCOI-2  laevCOI-1 | KJ943548  KJ943549  KJ943550 | laev18-1  laev18-1  laev18-1 |
|  | Lyutoga River, Sakhalin Island, Russia | 46°46’29” N,  142°26’21” E | 98-8  98-34  98-36 | IEPN d0077/8  IEPN d0077/34  IEPN d0077/36 | KJ161509  KJ161510  KJ161511 | laevCOI-5  laevCOI-6  laevCOI-4 | KJ957140  --  -- | laev18-1  --  -- |
|  | Golovnina River, Kunashir Island, Kurile Islands, Russia | 43°44’57” N,  145°30’25” E | 264-12  264-13  264-15 | IEPN d0044/12  IEPN d0044/13  IEPN d0044/15 | KJ161506  KJ161507  KJ161508 | laevCOI-7  laevCOI-7  laevCOI-7 | KJ943547  --  -- | laev18-1  --  -- |
|  | Serebryanka River, Kunashir Island, Kurile Islands, Russia | 44°03’18” N,  145°51’15” E | 263-17  263-19  263-21 | IEPN d0022/17  IEPN d0022/19  IEPN d0022/21 | KJ161497  KJ161498  KJ161499 | laevCOI-8  laevCOI-8  laevCOI-9 | --  --  -- | --  --  -- |
|  | Sennaya River, Kunashir Island, Kurile Islands, Russia | 43°44’56” N,  145°27’57” E | 260-1  259-22  259-23 | IEPN d0036/1  IEPN d0036/22  IEPN d0036/23 | KJ161504  KJ161500  KJ161505 | laevCOI-9  laevCOI-8  laevCOI-9 | --  KJ943546  -- | --  laev18-1  -- |
|  | Rikorda River, Kunashir Island, Kurile Islands, Russia | 43°44’58” N,  145°31’33” E | 261-27  261-28  261-29 | IEPN d0034/27  IEPN d0034/28  IEPN d0034/29 | KJ161501  KJ161502  KJ161503 | laevCOI-8  laevCOI-8  laevCOI-7 | --  --  -- | --  --  -- |
