# Supplementary material for: Taxonomy and Distribution of Freshwater Pearl Mussels (Unionoida: Margaritiferidae) of the Russian Far East
Source: PLoS One. 2015 May 26;10(5):e0122408. doi: 10.1371/journal.pone.0122408 (PMC4444039; doi:10.1371/journal.pone.0122408)
Supplement: S2 Table — (DOC) [file pone.0122408.s002.doc]

**Table S2. List of known localities of *Margaritifera dahurica* (Middendorff, 1850).**

| **No** | **River (stream)** | **River basin** | **Region** | **Year of occurrence** | **Status of record** | **Collector** | **Number of specimens** | **Locality coordinates** | | **Reference** |
| --- | --- | --- | --- | --- | --- | --- | --- | --- | --- | --- |
|  |  |  |  |  |  |  |  | **N** | **E** |  |
| 1 | Arkhara River | Amur River => Tatarsky strait (Okhotsk Sea) | Amur Oblast, Russia | 2008 | Viable population | A. Bylkov | 1 | 49°28'32" | 130°22'41" | [1] |
| 2 | Arkhara River | Amur River => Tatarsky strait (Okhotsk Sea) | Amur Oblast, Russia | 2008 | Viable population | A. Bylkov | 2 | 49°44'44" | 130°42'20" | [1] |
| 3 | Tanakan River | Arkhara River => Amur River => Tatarsky strait (Okhotsk Sea) | Amur Oblast, Russia | 2008 | Viable population | A. Bylkov | 1 | 49°31'30" | 130°37'13" | [1] |
| 4 | Argi River | Zeya River => Amur River => Tatarsky strait (Okhotsk Sea) | Amur Oblast, Russia | 1971 | Old occurrence | Sanaev | 4 | 54°39'47" | 129°06'27" | ZISP |
| 5 | Sungari River | Amur River River => Tatarsky strait (Okhotsk Sea) | China | 1925 | Old occurrence | V. Velichkovsky | 2 | 45°58'46" | 129°12'26" | ZISP |
| 6 | Iska River | Schastya Bay (Okhotsk Sea) | Khabarovsky kray, Russia | 1937 | Old occurrence | A. Taranets | 1 | 53°25'32" | 140°53'44" | ZISP |
| 7 | Bureya River | Amur River => Tatarsky strait (Okhotsk Sea) | Khabarovsky kray, Russia | 1976 | Old occurrence | V. Bogatov | 1 | 50°38'15" | 131°42'18" | ZISP |
| 8 | Bureya River | Amur River => Tatarsky strait (Okhotsk Sea) | Khabarovsky kray, Russia | 1980 | Old occurrence | M. Zatravkin | 9 | 50°47'24" | 132°02'19" | ZISP |
| 9 | Kur River | Tunguska River =>Amur River => Tatarsky strait (Okhotsk Sea) | Khabarovsky kray, Russia | 1953 | Old occurrence | V. Levanidov | 4 | 48°50'43" | 134°18'17" | ZISP |
| 10 | Belaya River | Chirka River =>Amur River => Tatarsky strait (Okhotsk Sea) | Khabarovsky kray, Russia | 1985-1986, 1989, 2002 | Viable population | A. Dolgih, I. Moskvicheva | 11 | 48°11'22" | 134°56'14" | ZISP |
| 11 | Nyura River | Dgzalunskoe Lake =>Amur River => Tatarsky strait (Okhotsk Sea) | Khabarovsky kray, Russia | 1930s | Old occurrence | A. Buldovsky | No data | 49°48'10" | 137°05'27" | [2] |
| 12 | El'ban (Ul'binka) River | Ommi Lake =>Amur River => Tatarsky strait (Okhotsk Sea) | Khabarovsky kray, Russia | 1930s | Old occurrence | A. Buldovsky | No data | 49°59'43" | 136°35'19" | [2] |
| 13 | Birakan River | Tunguzka River =>Amur River => Tatarsky strait (Okhotsk Sea) | Khabarovsky kray, Russia | 1931-1932 | Old occurrence | No data | No data | 49°07'30" | 134°28'50" | [2] |
| 14 | Khor River | Ussuri River =>Amur River => Tatarsky strait (Okhotsk Sea) | Khabarovsky kray, Russia | 1974 | Old occurrence | I. Moskvicheva | 6 | 47°41'22" | 135°42'10" | ZISP |
| 15 | Khor River | Ussuri River =>Amur River => Tatarsky strait (Okhotsk Sea) | Khabarovsky kray, Russia | 1930 | Old occurrence | B. Shtegman | 1 | 47°46'13" | 135°36'13" | ZISP |
| 16 | Khor River | Ussuri River =>Amur River => Tatarsky strait (Okhotsk Sea) | Khabarovsky kray, Russia | 1954 | Old occurrence | V. Levanidov | 1 | 47°54'03" | 135°12'54" | ZISP |
| 17 | Onon River | Shilka River =>Amur River => Tatarsky strait (Okhotsk Sea) | Mongolia | 1951 | Old occurrence | Anudarin | 1 | 49°21'51" | 112°12'55" | ZISP |
|  | Onon River | Shilka River =>Amur River => Tatarsky strait (Okhotsk Sea) | Mongolia | 1972 | Old occurrence | E. Devyatkin | 2 | 49°21'51" | 112°12'55" | ZISP |
| 18 | Ussuri River | Amur River => Tatarsky strait (Okhotsk Sea) | Primorsky kray, Russia | 1931 | Old occurrence | A. Buldovsky | No data | 44°31'05" | 134°02'50" | [2] |
|  | Pavlovka (Fudzin) River | Ussuri River =>Amur River => Tatarsky strait (Okhotsk Sea) | Primorsky kray, Russia | 1932 | Old occurrence | A. Buldovsky | No data | 44°23'49" | 134°04'00" | [2] |
|  | Pavlovka (Fudzin) River | Ussuri River =>Amur River => Tatarsky strait (Okhotsk Sea) | Primorsky kray, Russia | 1931 | Old occurrence | A. Buldovsky | No data | 44°24'20" | 134°13'42" | [2] |
| 19 | Ussuri River* | Amur River => Tatarsky strait (Okhotsk Sea) | Primorsky kray, Russia | 2012 | Viable population | Y. Bespalaya et al. | 20 | 43°51'14" | 133°52'29" | our field data: INEP [3] |
|  | Egerskaya (Sandagou) River | Ussuri River =>Amur River => Tatarsky strait (Okhotsk Sea) | Primorsky kray, Russia | 1931 | Old occurrence | A. Buldovsky | No data | 43°46'19" | 133°49'12" | [2] |
|  | Pravaya Poperechka (Sinancha) River | Ussuri River =>Amur River => Tatarsky strait (Okhotsk Sea) | Primorsky kray, Russia | 1931 | Old occurrence | A. Buldovsky | No data | 43°43'05" | 133°52'23" | [2] |
| 20 | Ussuri River* | Amur River => Tatarsky strait (Okhotsk Sea) | Primorsky kray, Russia | 2012 | Viable population | Y. Bespalaya et al. | 20 | 44°04'52" | 133°50'43" | our field data: INEP [3] |
|  | Ussuri River | Amur River => Tatarsky strait (Okhotsk Sea) | Primorsky kray, Russia | 1931 | Old occurrence | A. Buldovsky | No data | 43°59'57" | 133°54'22" | [2] |
|  | Ussuri River | Amur River => Tatarsky strait (Okhotsk Sea) | Primorsky kray, Russia | 1931 | Old occurrence | A. Buldovsky | No data | 44°07'54" | 133°51'30" | [2] |
|  | Ussuri River | Amur River => Tatarsky strait (Okhotsk Sea) | Primorsky kray, Russia | 1931 | Old occurrence | A. Buldovsky | No data | 44°13'36" | 133°48'60" | [2] |
|  | Ussuri River | Amur River => Tatarsky strait (Okhotsk Sea) | Primorsky kray, Russia | 1931 | Old occurrence | A. Buldovsky | No data | 44°15'44" | 133°52'48" | [2] |
|  | Matveevka (Tabakheza) River | Ussuri River =>Amur River => Tatarsky strait (Okhotsk Sea) | Primorsky kray, Russia | 1931 | Old occurrence | A. Buldovsky | No data | 44°12'09" | 133°47'16" | [2] |
| 21 | Peschernaya (Kulumbe) River | Japan Sea | Primorsky kray, Russia | 1931 | Old occurrence | A. Buldovsky | No data | 45°58'40" | 137°18'48" | [2] |
| 22 | Ilystaya River* | Khanka Lake (Ussuri River) =>Amur River => Tatarsky strait (Okhotsk Sea) | Primorsky kray, Russia | 2012 | Viable population | Y. Bespalaya et al. | 20 | 43°51'16" | 132°29'50" | our field data: INEP [3] |
| 23 | Komissarovka River | Khanka Lake (Ussuri River) =>Amur River => Tatarsky strait (Okhotsk Sea) | Primorsky kray, Russia | 1998 | Old occurrence | L. Prozorova | 1 | 44°45'25" | 131°25'29" | ZISP |
| 24 | Komissarovka River | Khanka Lake (Ussuri River) =>Amur River => Tatarsky strait (Okhotsk Sea) | Primorsky kray, Russia | 1986 | Old occurrence | V. Bogatov | 1 | 44°54'10" | 131°35'30" | ZISP |
|  | Komissarovka River | Khanka Lake (Ussuri River) =>Amur River => Tatarsky strait (Okhotsk Sea) | Primorsky kray, Russia | 1986 | Old occurrence | L. Prozorova | 1 | 44°55'56" | 131°37'36" | ZISP |
| 25 | Monastyrka Channel | Khanka Lake (Ussuri River) =>Amur River => Tatarsky strait (Okhotsk Sea) | Primorsky kray, Russia | 1912 | Old occurrence | A. Emelyanov | 1 | 44°11'13" | 132°29'40" | ZISP |
| 26 | Odarka River | Khanka Lake (Ussuri River) =>Amur River => Tatarsky strait (Okhotsk Sea) | Primorsky kray, Russia | 1911 | Old occurrence | A. Cherskiy | 9 | 44°37'11" | 133°05'07" | ZISP |
| 27 | Komarovka River | Razdol'naya River => Japan Sea | Primorsky kray, Russia | 1946-1947, 1972, 1999 | Old occurrence | I. Licharev, A. Tolstikov, L. Prozorova | 21 | 43°38'31" | 132°20'45" | ZISP |
| 28 | Komarovka River* | Razdol'naya River => Japan Sea | Primorsky kray, Russia | 2012 | Viable population | Y. Bespalaya et al. | 18 | 43°38'21" | 132°09'41" | our field data: INEP [3] |
| 29 | Arsen'evka (Daubikhe) River | Ussuri River =>Amur River => Tatarsky strait (Okhotsk Sea) | Primorsky kray, Russia | 1930s | Old occurrence | A. Buldovsky | No data | 44°10'04" | 133°13'58" | [2] |
| 30 | Arsen'evka River | Ussuri River =>Amur River => Tatarsky strait (Okhotsk Sea) | Primorsky kray, Russia | 1926 | Old occurrence | A. D'yakonov, P. Filip'ev | 1 | 44°24'37" | 133°30'00" | ZISP |
| 31 | Bolshaya Ussurka River | Ussuri River =>Amur River => Tatarsky strait (Okhotsk Sea) | Primorsky kray, Russia | 1988 | Old occurrence | V. Bogatov | 1 | 45°34'43" | 135°19'05" | ZISP |
| 32 | Bol'shaya Ussurka River | Ussuri River =>Amur River => Tatarsky strait (Okhotsk Sea) | Primorsky kray, Russia | 1931 | Old occurrence | A. Buldovsky | No data | 45°58'24" | 133°40'28" | [2] |
| 33 | Malinovka (Vaku) River | Ussuri River =>Amur River => Tatarsky strait (Okhotsk Sea) | Primorsky kray, Russia | 1931 | Old occurrence | A. Buldovsky | No data | 45°06'59" | 134°24'53" | [2] |
| 34 | Bira River | Amur River => Tatarsky strait (Okhotsk Sea) | The Jewish Autonomous Region, Russia | 1982 | Old occurrence | I. Moskvicheva | 5 | 48°59'28" | 132°26'59" | ZISP |
| 35 | Argun' River | Amur River => Tatarsky strait (Okhotsk Sea) | Zabaikalsky kray, Russia | 1973 | Old occurrence | A. Pavlov | 1 | 50°33'25" | 119°14'55" | ZISP |
| 36 | Argun' River | Amur River => Tatarsky strait (Okhotsk Sea) | Zabaikalsky kray, Russia | XIXth cent. | Old occurrence | No data | 1 | 53°19'21" | 121°26'33" | ZISP (holotype: no. 7a) |
| 37 | Budumkan River | Argun' River =>Amur River => Tatarsky strait (Okhotsk Sea) | Zabaikalsky kray, Russia | 2004 | Viable population | I. Mikheev | 2 | 52°40′12″ | 119°42′16″ | our field data: INREC |
| 38 | Gazimur River | Argun' River =>Amur River => Tatarsky strait (Okhotsk Sea) | Zabaikalsky kray, Russia | XIXth cent. | Old occurrence | No data | 1 | 52°03'57" | 118°53'33" | ZISP |
| 39 | Olenguy River* | Ingoda River => Shilka River =>Amur River => Tatarsky strait (Okhotsk Sea) | Zabaikalsky kray, Russia | 2010 | Viable population | O. Klishko | 1 | 51°40′23″ | 113°10′12″ | our field data: INREC |
| 40 | Arey Lake | Ingoda River (ancient connection) => Shilka River =>Amur River => Tatarsky strait (Okhotsk Sea) | Zabaikalsky kray, Russia | 2004 | Viable population | O. Klishko | 13 | 50°59′23″ | 111°14′42″ | our field data: ZISP & INREC |
| 41 | Ingoda River | Shilka River =>Amur River => Tatarsky strait (Okhotsk Sea) | Zabaikalsky kray, Russia | 2005 | Viable population | O. Klishko | 18 | 52°10′15″ | 113°10′16″ | our field data: ZISP & INREC |
| 42 | Ingoda River* | Shilka River =>Amur River => Tatarsky strait (Okhotsk Sea) | Zabaikalsky kray, Russia | 2010 | Viable population | O. Klishko | 1 | 51°20′12″ | 112°32′36″ | our field data: INREC |
| 43 | Ingoda River* | Shilka River =>Amur River => Tatarsky strait (Okhotsk Sea) | Zabaikalsky kray, Russia | 2009 | Viable population | O. Klishko | 1 | 51°32′14″ | 112°54′30″ | our field data: INREC |
| 44 | Ingoda River* | Shilka River =>Amur River => Tatarsky strait (Okhotsk Sea) | Zabaikalsky kray, Russia | 2011 | Viable population | O. Klishko | 3 | 51°59′29″ | 113°26′54″ | our field data: INREC |
| 45 | Ingoda River* | Shilka River =>Amur River => Tatarsky strait (Okhotsk Sea) | Zabaikalsky kray, Russia | 2010 | Viable population | O. Klishko | 3 | 51°59′60″ | 113°20′55″ | our field data: INREC |
| 46 | Nercha River* | Shilka River =>Amur River => Tatarsky strait (Okhotsk Sea) | Zabaikalsky kray, Russia | 2010 | Viable population | O. Klishko | 1 | 52°01′34″ | 116°28′32″ | our field data: INREC |
| 47 | Onon River | Shilka River =>Amur River => Tatarsky strait (Okhotsk Sea) | Zabaikalsky kray, Russia | 2009 | Viable population | O. Klishko | 1 | 50°08'24" | 113°02'28" | our field data: INREC |
| 48 | Onon River | Shilka River =>Amur River => Tatarsky strait (Okhotsk Sea) | Zabaikalsky kray, Russia | 2004 | Viable population | D. Matafonov | 2 | 51°18′20″ | 116°06′14″ | our field data: ZISP & IBSS |
| 49 | Onon River | Shilka River =>Amur River => Tatarsky strait (Okhotsk Sea) | Zabaikalsky kray, Russia | 2004 | Viable population | D. Matafonov | 2 | 51°18′36″ | 116°20′10″ | our field data: ZISP |
| 50 | Onon River* | Shilka River =>Amur River => Tatarsky strait (Okhotsk Sea) | Zabaikalsky kray, Russia | 2010 | Viable population | O. Klishko | 8 | 49°40′01″ | 112°38′14″ | our field data: INREC |

* - species identification was verified by COI data (see Table S1).

** - river where locality of M. dahurica were detected

# References

# Sayenko EM, Balan IV. New data on large bivalves (Margaritiferidae, Unionidae) of the Khingansky Reserve and adjacent areas (Amurskaya Territory) [in Russian]. The Bulletin of the Russian Far East Malacological Society. 2009; 13: 63–69.

# Buldovsky AT. About harvested freshwater mussels of the Soviet Far East [in Russian]. Proceedings of the Far Eastern Branch of the USSR Academy of Sciences. 1935; 12: 39–65.

# Bespalaya Y, Bolotov I, Vikhrev I, Kondakov A, Gofarov M, et al. Ecology and current status of the freshwater pearl mussels *Margaritifera dahurica* (Middendorff, 1850) and *Margaritifera laevis* (Haas, 1910) (Bivalvia: Unionoidea) in the Far East of Russia. Açoreana: Revista de Estudos Açoreanos (World Congress of Malacology, Book of Abstracts); 2013. Supl. 8: 204.
