# Supplementary material for: Taxonomy and Distribution of Freshwater Pearl Mussels (Unionoida: Margaritiferidae) of the Russian Far East
Source: PLoS One. 2015 May 26;10(5):e0122408. doi: 10.1371/journal.pone.0122408 (PMC4444039; doi:10.1371/journal.pone.0122408)
Supplement: S3 Table — (DOC) [file pone.0122408.s003.doc]

**Table S3.** List of known localities of *Margaritifera middendorffi* (Rosén, 1926) and *Margaritifera togakushiensis* (Kondo and Kobayashi, 2005).

| **No** | **River (stream)** | **River basin** | **Region** | **Year of occurrence** | **Status of record** | **Collector** | **Number of specimens** | **Locality coordinates** | | **Reference** |
| --- | --- | --- | --- | --- | --- | --- | --- | --- | --- | --- |
|  |  |  |  |  |  |  |  | **N** | **E** |  |
| *Margaritifera middendorffi* (Rosén, 1926) | | | | | | | | | | |
| 1 | Golygina River | Okhotsk Sea | Kamchatsky kray, Russia | 1908-1909 | Old occurrence | Ruvinsky | 8 | 51°52'31" | 156°37'20" | ZISP; [1] |
| 2 | Khlebnaya River | Okhotsk Sea | Kamchatsky kray, Russia | XXth cent. | Old occurrence | No data | No data | 56°55'57" | 156°53'20" | [1] |
| 3 | Kol' River | Okhotsk Sea | Kamchatsky kray, Russia | XXth cent. | Old occurrence | No data | No data | 53°49'31" | 156°04'59" | [1] |
| 4 | Mekeshino Lake | Okhotsk Sea | Kamchatsky kray, Russia | 1847 | Old occurrence | I.G. Voznesensky | 6 | 50°55'25" | 156°42'57" | ZISP: include Rozen's paralectotypes |
| 5 | Nachilova River* | Okhotsk Sea | Kamchatsky kray, Russia | 2012 | Viable population | Y. Bespalaya et al. | 58 | 52°53'06" | 156°26'54" | our field data: INEP |
| 6 | Opala River | Okhotsk Sea | Kamchatsky kray, Russia | XXth cent. | Old occurrence | No data | No data | 52°10'15" | 156°34'29" | [1] |
| 7 | Vorovskaya River | Okhotsk Sea | Kamchatsky kray, Russia | XXth cent. | Old occurrence | No data | No data | 54°20'22" | 156°00'60" | [1] |
| 8 | Paratunka River | Pacific Ocean | Kamchatsky kray, Russia | 1964 | Old occurrence | G.P. Borzunov | 1 | 53°01'08" | 158°17'48" | ZISP |
| 9 | Golovnina River*, ** | Pacific Ocean | Kunashir Island, Russia | 1971, 1972, 2011 | Viable population | Y. Bespalaya et al., A.A. Shileiko, B.I. Sirenko | 20 | 43°44'57" | 145°30'25" | our field data: INEP; ZISP |
|  | Sennaya River*, ** | Pacific Ocean | Kunashir Island, Russia | 2011 | Viable population | Y. Bespalaya et al. | 20 | 43°44'56" | 145°27'57" | our field data: INEP |
| 10 | Serebryanka River*, ** | Pacific Ocean | Kunashir Island, Russia | 2011 | Viable population | Y. Bespalaya et al. | 20 | 44°03'18" | 145°51'15" | our field data: INEP |
| 11 | Tym' River*, ** | Pacific Ocean | Sakhalin Island, Russia | 2012 | Viable population | Y. Bespalaya et al. | 20 | 50°54'45" | 142°39'48" | our field data: INEP |
|  | Confluence of Adamka and Armudanka rivers* | Tym' River (Pacific Ocean) | Sakhalin Island, Russia | 2012 | Viable population | Y. Bespalaya et al. | 20 | 50°49'42" | 142°33'18" | our field data: INEP |
|  | Voskresenovka River* | Tym' River (Pacific Ocean) | Sakhalin Island, Russia | 2012 | Viable population | Y. Bespalaya et al. | 20 | 50°54'10" | 142°41'04" | our field data: INEP |
| 12 | No name stream | Okhotsk Sea | Shumshu Island, Sakhalin Oblast, Russia | No data | Old occurrence | No data | No data | 50°45'50" | 156°15'19" | [1] |
| *Margaritifera togakushiensis* (Kondo & Kobayashi, 2005) | | | | | | | | | | |
| 13 | Dagi River | Pacific Ocean | Sakhalin Island, Russia | 2013 | Viable population | Yu. Akiyama | 1 | 52°07'04" | 143°00'29" | [2] |
| 14 | Urashibetsugawa | Japan Sea | Hokkaido Island, Japan | No data | No data | No data | No data | 43°52'12" | 144°26'15" | [3] |
| 14 | Pemen River | Japan Sea | Hokkaido Island, Japan | No data | No data | No data | No data | 43°47'40" | 144°33'31" | [3] |
| 15 | Seventh Shigetaro River | Pacific Ocean | Hokkaido Island, Japan | No data | No data | No data | No data | 43°27'1" | 145°7'31" | [3] |
| 16 | Numakawa River | Pacific Ocean | Hokkaido Island, Japan | No data | No data | No data | No data | 43°7'29" | 145°4'4" | [3] |
| 16 | No name stream | Pacific Ocean | Hokkaido Island, Japan | No data | No data | No data | No data | 43°2'22" | 144°52'52" | [3] |
| 17 | Ruon'nenai River | Pacific Ocean | Hokkaido Island, Japan | No data | No data | No data | No data | 43°7'32" | 144°3'31" | [3] |
| 18 | Ishikarigawa River | Japan Sea | Hokkaido Island, Japan | No data | No data | No data | No data | 43°22'29" | 142°19'5" | [3] |
| 19 | Chopetan River | Japan Sea | Hokkaido Island, Japan | No data | No data | No data | No data | 43°16'3" | 140°37'33" | [3] |
| 20 | Akagawa River | Pacific Ocean | Honshu Island, Japan | No data | No data | No data | No data | 39°57'36" | 141°3'33" | [3] |
| 21 | Saksa River | Japan Sea | Honshu Island, Japan | 2005 | Viable population | Yu. Akiyama | No data | 36°36'52" | 138°14'24" | [4] |

* - species identification was verified by COI data (see Table S1).

** - these rivers also inhabit *M. laevis*.

**References**

1. Kurenkov II. On distribution of the Kamchatka freshwater pearl mussel [in Russian]. Questions of Kamchatka geography. 1966; 4: 110–112.

# Akiyama B, Kimura R, Nomoto K, Usui T, Machida Y. New record of the freshwater pearl mussel *Margaritifera togakushiensis* from northern Sakhalin, the Russian Far East. Venus. 2013; 71: 191–198.

1. Uchiyama R, Kondo T. Margaritifera laevis, M. togakushiensis (Margaritiferidae). In: Animal Distribution Atlas of Japan. The Biodiversity Center of Japan, the Nature Conservation Bureau, the Ministry of the Environment, Japan; 2010. p. 43
2. Kondo T, Kobayashi O. Revision of the Genus *Margaritifera* (Bivalvia: Margaritiferidae) of Japan, with description of a New Species. Venus. 2005; 64: 135–140.
