# Supplementary material for: Taxonomy and Distribution of Freshwater Pearl Mussels (Unionoida: Margaritiferidae) of the Russian Far East
Source: PLoS One. 2015 May 26;10(5):e0122408. doi: 10.1371/journal.pone.0122408 (PMC4444039; doi:10.1371/journal.pone.0122408)
Supplement: S4 Table — (DOC) [file pone.0122408.s004.doc]

**Table S4.** List of known localities of *Margaritifera laevis* (Haas, 1910).

| **No** | **River (stream)** | **River basin** | **Region** | **Year of occurrence** | **Status of record** | **Collector** | **Number of specimens** | **Locality coordinates** | | **Reference** |
| --- | --- | --- | --- | --- | --- | --- | --- | --- | --- | --- |
|  |  |  |  |  |  |  |  | **N** | **E** |  |
| 1 | No name stream | Kurilka River => Okhotsk Sea | Iturup Island, Kurile Arhipelago, Russia | 1964 | Old occurrence | Kononenko | 1 | 45°13'24" | 147°53'18" | ZISP |
| 2 | Golovnina River*, ** | Pacific Ocean | Kunashir Island, Russia | 2011 | Viable population | Y. Bespalaya et al. | 20 | 43°44'57" | 145°30'25" | our field data: INEP |
| 2 | Rikorda River | Pacific Ocean | Kunashir Island, Russia | 2011 | Viable population | Y. Bespalaya et al. | 20 | 43°44'58" | 145°31'33" | our field data: INEP |
| 2 | Sennaya River*, ** | Pacific Ocean | Kunashir Island, Russia | 2011 | Viable population | Y. Bespalaya et al. | 20 | 43°44'56" | 145°27'57" | our field data: INEP |
| 3 | Serebryanka River*, ** | Pacific Ocean | Kunashir Island, Russia | 2011 | Viable population | Y. Bespalaya et al. | 20 | 44°03'18" | 145°51'15" | our field data: INEP |
| 4 | Lyutoga River | Okhotsk Sea | Sakhalin Island, Russia | 2011-2012 | Viable population | Y. Bespalaya et al. | 20 | 46°46'29" | 142°26'21" | our field data: INEP |
| 5 | Poronay River | Okhotsk Sea | Sakhalin Island, Russia | 1997 | Old occurrence | V.N. Nikitin | 10 | 50°33'02" | 142°46'04" | ZISP |
| 6 | Tym' River*, ** | Okhotsk Sea | Sakhalin Island, Russia | 1934 | Old occurrence | A. Taranets | 27 | 50°54'45" | 142°39'48" | our field data: ZISP & INEP |
| 7 | Dagi River | Pacific Ocean | Sakhalin Island, Russia | 2013 | Viable population | Yu. Akiyama | 25 | 52°07'04" | 143°00'29" | [1] |
| 8 | No name river near Anama (Crab) bay | Okhotsk Sea | Shikotan Island, Kurile Arhipelago, Russia | 1949 | Old occurrence | E.F. Gur'yanova | 2 | 43°48'49" | 146°44'57" | ZISP |
| 9 | Kusiro River | Pacific Ocean | Hokkaido Island, Japan | 1984 | Old occurrence | T. Habe | 2 | 43° 01'04" | 144°25'47" | ZISP |
| 10 | Kimoma Numakawa River | Japan Sea | Hokkaido Island, Japan | No data | No data | No data | No data | 45°17' N | 142°11'8" | [2] |
| 10 | Nitatoroomanai River | Japan Sea | Hokkaido Island, Japan | No data | No data | No data | No data | 45°17'21" | 141°56'21" | [2] |
| 10 | Menashibetsu nine-wire River | Japan Sea | Hokkaido Island, Japan | No data | No data | No data | No data | 45°12'40." | 141°55'57" | [2] |
| 10 | Seventeen Route River | Japan Sea | Hokkaido Island, Japan | No data | No data | No data | No data | 45°7'7" | 142°3'41" | [2] |
| 10 | Sarufutsugawa River | Japan Sea | Hokkaido Island, Japan | No data | No data | No data | No data | 45°7'25" | 142°11'51" | [2] |
| 11 | Shunbetsugawa River | Pacific Ocean | Hokkaido Island, Japan | No data | No data | No data | No data | 43°28'31" | 145°0'20" | [2] |
| 12 | No. 24 River | Pacific Ocean | Hokkaido Island, Japan | No data | No data | No data | No data | 43°17'20" | 144°41'31" | [2] |
| 12 | Kushirogawa River | Pacific Ocean | Hokkaido Island, Japan | No data | No data | No data | No data | 43°12'37" | 144°33'39" | [2] |
| 13 | Oborogawa River | Pacific Ocean | Hokkaido Island, Japan | No data | No data | No data | No data | 43°3'5" | 144°45'32" | [2] |
| 14 | No name stream | Pacific Ocean | Hokkaido Island, Japan | No data | No data | No data | No data | 41°57'59" | 143°11'51" | [2] |
| 15 | Abiragawa River | Pacific Ocean | Hokkaido Island, Japan | No data | No data | No data | No data |  |  | [2] |
| 15 | Abiragawa River | Pacific Ocean | Hokkaido Island, Japan | No data | No data | No data | No data | 42°51'56" | 141°56'9" | [2] |
| 15 | Ishikarigawa River | Pacific Ocean | Hokkaido Island, Japan | No data | No data | No data | No data | 42°52'24" | 141°48'42" | [2] |
| 15 | Abiragawa River | Pacific Ocean | Hokkaido Island, Japan | No data | No data | No data | No data | 42°52'46" | 141°40'50" | [2] |
| 15 | Otaru River | Pacific Ocean | Hokkaido Island, Japan | No data | No data | No data | No data | 42°47'37" | 141°42'3" | [2] |
| 16 | Obopu River | Pacific Ocean | Hokkaido Island, Japan | No data | No data | No data | No data | 42°42'46" | 141°41'3" | [2] |
| 17 | Shakotan River | Japan Sea | Hokkaido Island, Japan | No data | No data | No data | No data | 43°17'46" | 140°29'19" | [2] |
| 18 | Osan'nai first River | Japan Sea | Hokkaido Island, Japan | No data | No data | No data | No data | 42°52'33" | 140°26'39" | [2] |
| 18 | The right Kadonosawa River | Japan Sea | Hokkaido Island, Japan | No data | No data | No data | No data | 42°47'15" | 140°25'46" | [2] |
| 18 | Nishinosawa River | Japan Sea | Hokkaido Island, Japan | No data | No data | No data | No data | 42°47'26" | 140°33'54" | [2] |
| 19 | Tomeno Sogo River | Pacific Ocean | Hokkaido Island, Japan | No data | No data | No data | No data | 42°3'28" | 140°41'32" | [2] |
| 20 | No name stream | Pacific Ocean | Honshu Island, Japan | No data | No data | No data | No data | 41°17'22" | 141°10'49" | [2] |
| 20 | No name stream | Pacific Ocean | Honshu Island, Japan | No data | No data | No data | No data | 41°17'43" | 141°18'53" | [2] |
| 21 | Yachinaka River | Pacific Ocean | Honshu Island, Japan | No data | No data | No data | No data | 40°7'32" | 141°48'35" | [2] |
| 21 | Akkagawa River | Pacific Ocean | Honshu Island, Japan | No data | No data | No data | No data | 40°2'2" | 141°48'25" | [2] |
| 21 | Sawa River | Pacific Ocean | Honshu Island, Japan | No data | No data | No data | No data | 39°57'16" | 141°41'24" | [2] |
| 21 | Omotogawa River | Pacific Ocean | Honshu Island, Japan | No data | No data | No data | No data | 39°52'24" | 141°48'48" | [2] |
| 22 | Kitakamigawa River | Pacific Ocean | Honshu Island, Japan | No data | No data | No data | No data | 39°48'10" | 141°2'32" | [2] |
| 23 | Omonogawa River | Japan Sea | Honshu Island, Japan | No data | No data | No data | No data | 39°37'15" | 140°10'39" | [2] |
| 24 | Yamafunyu River | Pacific Ocean | Honshu Island, Japan | No data | No data | No data | No data | 37°52'28" | 140°41'32" | [2] |
| 24 | Manogawa River | Pacific Ocean | Honshu Island, Japan | No data | No data | No data | No data | 37°42'32" | 140°40'58" | [2] |
| 25 | Hisogawa River | Pacific Ocean | Honshu Island, Japan | No data | No data | No data | No data | 37°37'15" | 140°48'27" | [2] |
| 25 | Ukedogawa River | Pacific Ocean | Honshu Island, Japan | No data | No data | No data | No data | 37°32'35" | 140°48'55" | [2] |
| 25 | Kawabusa River | Pacific Ocean | Honshu Island, Japan | No data | No data | No data | No data | 37°32'26" | 140°56'28" | [2] |
| 25 | Takasegawa River | Pacific Ocean | Honshu Island, Japan | No data | No data | No data | No data | 37°27'47" | 140°55'48" | [2] |
| 25 | Ishiguro River | Pacific Ocean | Honshu Island, Japan | No data | No data | No data | No data | 37°27'7" | 140°47'59" | [2] |
| 26 | Komatsugawa River | Pacific Ocean | Honshu Island, Japan | No data | No data | No data | No data | 37°2'12" | 140°33'59" | [2] |
| 26 | Kawakamigawa River | Pacific Ocean | Honshu Island, Japan | No data | No data | No data | No data | 36°57'5" | 140°26'22" | [2] |
| 26 | Tagawa River | Pacific Ocean | Honshu Island, Japan | No data | No data | No data | No data | 36°52'0" | 140°25'35" | [2] |
| 26 | Samegawa River | Pacific Ocean | Honshu Island, Japan | No data | No data | No data | No data | 36°52'19" | 140°33'27" | [2] |
| 26 | Okitagawa River | Pacific Ocean | Honshu Island, Japan | No data | No data | No data | No data | 36°47'27" | 140°33'57" | [2] |
| 27 | Owasu River | Pacific Ocean | Honshu Island, Japan | No data | No data | No data | No data | 36°57'20" | 140°11'11" | [2] |
| 27 | Nakagawa River | Pacific Ocean | Honshu Island, Japan | No data | No data | No data | No data | 37°2'2" | 140°3'34" | [2] |
| 27 | Makigawa River | Pacific Ocean | Honshu Island, Japan | No data | No data | No data | No data | 36°51'47" | 140°3'56" | [2] |
| 28 | Kanda River | Japan Sea | Honshu Island, Japan | No data | No data | No data | No data | 37°21'52" | 136°56'20" | [2] |
| 29 | Shinanogawa River | Japan Sea | Honshu Island, Japan | No data | No data | No data | No data | 36°47'14" | 138°2'57" | [2] |
| 30 | Nigorisawagawa River | Japan Sea | Honshu Island, Japan | No data | No data | No data | No data | 36°37'30" | 138°6'50" | [2] |
| 31 | Daigosawa River | Japan Sea | Honshu Island, Japan | No data | No data | No data | No data | 36°37'2" | 137°48'11" | [2] |
| 31 | Saksa River | Japan Sea | Honshu Island, Japan | 2005 | Viable population | Yu. Akiyama | No data | 36°31'56" | 137°48'9" | [2] |
| 32 | Arakigawa River | Pacific Ocean | Honshu Island, Japan | No data | No data | No data | No data | 36°12'12" | 137°18'59" | [2] |
| 32 | Cubu-Nogu River | Pacific Ocean | Honshu Island, Japan | 2005 | Viable population | Yu. Akiyama | No data | 36°12'17" | 137°11'23" | [2] |
| 32 | Kawakamigawa | Pacific Ocean | Honshu Island, Japan | No data | No data | No data | No data | 36°7'33" | 137°11'3" | [2] |
| 32 | Jinzu River | Pacific Ocean | Honshu Island, Japan | No data | No data | No data | No data | 36°7'13" | 137°19'9" | [2] |
| 32 | Mouth Arimichitani River | Pacific Ocean | Honshu Island, Japan | No data | No data | No data | No data | 36°2'9" | 137°18'52" | [2] |
| 32 | Kiso | Pacific Ocean | Honshu Island, Japan | No data | No data | No data | No data | 35°57'11" | 137°11'14" | [2] |
| 33 | Black dong River | Pacific Ocean | Honshu Island, Japan | No data | No data | No data | No data | 35°47'14" | 136°56'5" | [2] |
| 33 | Akatani River | Pacific Ocean | Honshu Island, Japan | No data | No data | No data | No data | 35°42'6" | 136°56'3" | [2] |
| 34 | Shinsakai River | Pacific Ocean | Honshu Island, Japan | No data | No data | No data | No data | 35°22'14" | 136°48'12" | [2] |
| 35 | Biwako River | Lake Biwa => Japan Sea | Honshu Island, Japan | No data | No data | No data | No data | 35°12'50" | 135°55'38" | [2] |
| 36 | Yura River | Japan Sea | Honshu Island, Japan | No data | No data | No data | No data | 35°17'41" | 135°18'50" | [2] |
| 37 | Daiya River | Japan Sea | Honshu Island, Japan | No data | No data | No data | No data | 34°41'50" | 133°41'43" | [2] |
| 38 | Takahashi River | Pacific Ocean | Honshu Island, Japan | No data | No data | No data | No data | 34°52'38" | 133°11'12" | [2] |
| 39 | Scioto River | Japan Sea | Honshu Island, Japan | No data | No data | No data | No data | 34°47'27" | 132°18'55" | [2] |
| 39 | Takiyamagawa River | Japan Sea | Honshu Island, Japan | No data | No data | No data | No data | 34°42'25" | 132°18'13" | [2] |
| 40 | Yawata Oikawa River | Pacific Ocean | Honshu Island, Japan | No data | No data | No data | No data | 34°22'26" | 132°11'25" | [2] |
| 40 | Kujima River | Pacific Ocean | Honshu Island, Japan | No data | No data | No data | No data | 34°17'47" | 132°11'24" | [2] |

* - species identification was verified by COI data (see Table S1).

** - these rivers also inhabit *M. middendorffi*.

**References**

# Akiyama B, Kimura R, Nomoto K, Usui T, Machida Y. New record of the freshwater pearl mussel *Margaritifera togakushiensis* from northern Sakhalin, the Russian Far East. Venus. 2013; 71: 191–198.

1. Uchiyama R, Kondo T. *Margaritifera laevis*, *M. togakushiensis* (Margaritiferidae). In: *Animal Distribution Atlas of Japan*. The Biodiversity Center of Japan, the Nature Conservation Bureau, the Ministry of the Environment, Japan; 2010. p. 43.
