# Supplementary material for: Taxonomy and Distribution of Freshwater Pearl Mussels (Unionoida: Margaritiferidae) of the Russian Far East
Source: PLoS One. 2015 May 26;10(5):e0122408. doi: 10.1371/journal.pone.0122408 (PMC4444039; doi:10.1371/journal.pone.0122408)
Supplement: S5 Table — (DOC) [file pone.0122408.s005.doc]

**Table S5.** Variance and significance tests results for mantle attachment scars density within three Margaritefirid species (*M. dahurica*, n – 20; *M. laevis*, n – 20; *M. middendorffi*, n – 20)

|  | F | Df | p |
| --- | --- | --- | --- |
| Levene’s test, from means |  |  | 0.0397 |
| Welch’s test | 1.43 | 16.38 | 0.257 |
