# Supplementary material for: Taxonomy and Distribution of Freshwater Pearl Mussels (Unionoida: Margaritiferidae) of the Russian Far East
Source: PLoS One. 2015 May 26;10(5):e0122408. doi: 10.1371/journal.pone.0122408 (PMC4444039; doi:10.1371/journal.pone.0122408)
Supplement: S6 Table — (DOC) [file pone.0122408.s006.doc]

**Table S6.** List of additional DNA sequences of *Margaritifera* spp. obtained from NCBI’s GenBank including accession number, taxon, specimen code, locality information and references

| Gene | Haplotype code | Acc. no. | Species | Specimen Voucher | Locality | References |
| --- | --- | --- | --- | --- | --- | --- |
| 18S | outgroup | KC429109 | *U. pictorum* | BivAToL-204 | River Thames, UK | [1] |
| 18S | mar18S01 | KC429348 | *M. margaritifera* | BivAToL-299 | Northern Ireland | [1] |
| 18S | mrr18S01 | AY579104 | *M. marrianae* | MCZ DNA100695 | Alabama, USA | [2] |
| 18S | fal18S01 | AY579098 | *M. falcata* | MCZ DNA100670 | Idaho, USA | [2] |
| 18S | aur18S01 | AY579097 | *M. auricularia* | MCZ DNA100674 | Spain | [2] |
| 18S | aur18S02 | AY579096 | *M. auricularia* | MCZ DNA100672 | Spain | [2] |
| 18S | mon18S01 | AY579105 | *C. monodonta* | MCZ DNA100863 | Missouri, USA | [2] |
| COI | outgroup | KC429109 | *U. pictorum* | BivAToL-204 | River Thames, UK | [1] |
| COI | falCOI01 | DQ272374 | *M. falcata* | 105BearCreek | Washington, USA | [3] |
| COI | falCOI02 | DQ272375 | *M. falcata* | 107BearCreek | Washington, USA | [3] |
| COI | falCOI03 | DQ272379 | *M. falcata* | 183YakimaRiver | Washington, USA | [3] |
| COI | falCOI04 | DQ272376 | *M. falcata* | 157PilchuckCreek | Washington, USA | [3] |
| COI | falCOI05 | DQ272377 | *M. falcata* | 158PilchuckCreek | Washington, USA | [3] |
| COI | falCOI06 | AY579128 | *M. falcata* | MCZ DNA100844 | Idaho, USA | [2] |
| COI | marCOI01 | AF303316 | *M. margaritifera* | FW1194-3 | Europe | [9] |
| COI | marCOI02 | AF303321 | *M. margaritifera* | FW1296-16 | Europe | [9] |
| COI | marCOI03 | AF303330 | *M. margaritifera* | FW1405-5 | Europe | [9] |
| COI | marCOI04 | AF303331 | *M. margaritifera* | FW1407-2 | Europe | [9] |
| COI | mroCOI01 | EU429685 | *M. marocana* | MNCN:N1271 | Abid, Imadahine, Morocco | [4] |
| COI | mroCOI02 | EU429680 | *M. marocana* | MNCN:N1266 | Abid, Imadahine, Morocco | [4] |
| COI | mroCOI03 | EU429678 | *M. marocana* | MNCN:N1254 | Oum Er Rbia, Morocco | [4] |
| COI | mroCOI04 | EU429677 | *M. marocana* | MNCN:N1252 | Oum Er Rbia, Morocco | [4] |
| COI | aurCOI01 | JX046574 | *M. auricularia* | MNHN-IM-2009-12611 | Vienne, France | [5] |
| COI | aurCOI02 | AF303310 | *M. auricularia* | FW1238-14 | Canal Imperial de Aragón  (Zaragoza, Spain) | [6] |
| COI | monCOI01] | KF647332 | *C. monodonta* | CM002G | Missouri, USA | [7] |

**References**

1. Sharma PP, Zardus JD, Boyle EE, Gonzalez VL, Jennings RM, McIntyre E, Wheeler WC, Etter RJ, Giribet G. Into the deep: a phylogenetic approach to the bivalve subclass Protobranchia. Mol Phylogenet Evol. 2013; 69: 188–204.
2. Huff SW, Campbell D, Gustafson DL, Lydeard C, Altaba CR, Giribet G. Investigations into the phylogenetic relationships of freshwater pearl mussels (Bivalvia: Margaritiferidae) based on molecular data: implications for their taxonomy and biogeography. J Mollus Stud. 2004; 70: 379–388.
3. Gustafson RG, Iwamoto EM. A DNA-based identification key to Pacific Northwest freshwater mussel glochidia: importance to salmonid and mussel conservation. Northwest Science. 2005; 79: 233–245.
4. Araujo R, Toledo C, Van Damme D, Ghamizi M, Machordom A. *Margaritifera marocana* (Pallary, 1918): a valid species inhabiting Moroccan rivers. J Mollus Stud. 2007; 75: 95–101.
5. Prie V, Puillandre N, Bouchet P (2012) Bad taxonomy can kill: molecular reevaluation of Unio mancus Lamarck, 1819 (Bivalvia: Unionidae) and its accepted subspecies. Knowledge and Management of Aquatic Ecosystems, 405, 08.
6. Machordom A, Araujo R, Erpenbeck D, Ramos M-A. Phylogeography and conservation genetics of endangered European Margaritiferidae (Bivalvia: Unionoidea). Biol J Linn Soc. 2003; 78: 235–252.
7. Inoue K, Monroe EM, Elderkin CL, Berg DJ. Phylogeographic and population genetic analyses reveal Pleistocene isolation followed by high gene flow in a wide ranging, but endangered, freshwater mussel. Heredity. 2013; 112: 282–290.
